# Walking on common ground: a cross-disciplinary scoping review on the clinical utility of digital mobility outcomes

**DOI:** 10.1038/s41746-021-00513-5

**Published:** 2021-10-14

**Authors:** Ashley Polhemus, Laura Delgado-Ortiz, Gavin Brittain, Nikolaos Chynkiamis, Francesca Salis, Heiko Gaßner, Michaela Gross, Cameron Kirk, Rachele Rossanigo, Kristin Taraldsen, Diletta Balta, Sofie Breuls, Sara Buttery, Gabriela Cardenas, Christoph Endress, Julia Gugenhan, Alison Keogh, Felix Kluge, Sarah Koch, M. Encarna Micó-Amigo, Corinna Nerz, Chloé Sieber, Parris Williams, Ronny Bergquist, Magda Bosch de Basea, Ellen Buckley, Clint Hansen, A. Stefanie Mikolaizak, Lars Schwickert, Kirsty Scott, Sabine Stallforth, Janet van Uem, Beatrix Vereijken, Andrea Cereatti, Heleen Demeyer, Nicholas Hopkinson, Walter Maetzler, Thierry Troosters, Ioannis Vogiatzis, Alison Yarnall, Clemens Becker, Judith Garcia-Aymerich, Letizia Leocani, Claudia Mazzà, Lynn Rochester, Basil Sharrack, Anja Frei, Milo Puhan, Mobilise-D

**Affiliations:** 1 Epidemiology, Biostatistics and Prevention Institute, University of Zurich, Zurich, Switzerland; 2 ISGlobal, Barcelona, Spain; 3 Universitat Pompeu Fabra, Barcelona, Spain; 4 CIBER Epidemiología y Salud Pública, Barcelona, Spain; 5 Department of Neuroscience and Sheffield NIHR Translational Neuroscience BRC, Sheffield Teaching Hospitals NHS Foundation Trust & University of Sheffield, Sheffield, England; 6 Department of Sport, Exercise and Rehabilitation, Faculty of Health and Life Sciences, Northumbria University Newcastle, Newcastle, UK; 7 Department of Biomedical Sciences, University of Sassari, Sassari, Italy; 8 Department of Molecular Neurology, University Hospital Erlangen, Erlangen, Germany; 9 Department of Clinical Gerontology, Robert-Bosch-Hospital, Stuttgart, Germany; 10 Translational and Clinical Research Institute, Faculty of Medical Sciences, Newcastle University, Newcastle upon Tyne, UK; 11 Department of Neuromedicine and Movement Science, Norwegian University of Science and Technology, Trondheim, Norway; 12 Department of Electronics and Telecommunications, Politecnico di Torino, Torino, Italy; 13 Department of Rehabilitation Sciences, KU Leuven, Leuven, Belgium; 14 Department of Respiratory Diseases, University hospitals Leuven, Leuven, Belgium; 15 National Heart and Lung Institute, Imperial College London, London, UK; 16 Insight Centre for Data Analytics, University College Dublin, Dublin, Ireland; 17 Department of Artificial Intelligence in Biomedical Engineering, Friedrich-Alexander-Universität Erlangen-Nürnberg (FAU), Erlangen, Germany; 18 Insigneo Institute, Department of Mechanical Engineering, University of Sheffield, Sheffield, UK; 19 Department of Neurology, University Medical Center Schleswig-Holstein, Kiel, Germany; 20 Department of Rehabilitation Sciences, Ghent University, Ghent, Belgium; 21 Department of Neurology, San Raffaele University, Milan, Italy

**Keywords:** Predictive markers, Geriatrics, Movement disorders, Respiratory tract diseases

## Abstract

Physical mobility is essential to health, and patients often rate it as a high-priority clinical outcome. Digital mobility outcomes (DMOs), such as real-world gait speed or step count, show promise as clinical measures in many medical conditions. However, current research is nascent and fragmented by discipline. This scoping review maps existing evidence on the clinical utility of DMOs, identifying commonalities across traditional disciplinary divides. In November 2019, 11 databases were searched for records investigating the validity and responsiveness of 34 DMOs in four diverse medical conditions (Parkinson’s disease, multiple sclerosis, chronic obstructive pulmonary disease, hip fracture). Searches yielded 19,672 unique records. After screening, 855 records representing 775 studies were included and charted in systematic maps. Studies frequently investigated gait speed (70.4% of studies), step length (30.7%), cadence (21.4%), and daily step count (20.7%). They studied differences between healthy and pathological gait (36.4%), associations between DMOs and clinical measures (48.8%) or outcomes (4.3%), and responsiveness to interventions (26.8%). Gait speed, step length, cadence, step time and step count exhibited consistent evidence of validity and responsiveness in multiple conditions, although the evidence was inconsistent or lacking for other DMOs. If DMOs are to be adopted as mainstream tools, further work is needed to establish their predictive validity, responsiveness, and ecological validity. Cross-disciplinary efforts to align methodology and validate DMOs may facilitate their adoption into clinical practice.

## Introduction

Physical mobility is an essential aspect of health. Mobility impairment is associated with reduced quality of life, falls, hospitalization, mortality, and other adverse events in many chronic conditions^1–7^. It is therefore unsurprising that people living with chronic conditions often rate physical mobility—and specifically walking ability—as one of the most important clinical outcomes^8–13^.

Traditional mobility measures include patient-reported outcomes (how well an *individual thinks* they can walk), objective clinical assessments (an *individual’s examined capacity* to walk), and subjective clinical assessments (how well a *clinician thinks* an individual can walk given a set of standard criteria). These measures can be subject to recall bias, Hawthorne effects, substantial training requirements, and ceiling or floor effects, among other limitations^14–20^. They are acquired infrequently and often conducted in clinical settings that rarely reflect the complex environmental determinants of real-world function, raising questions of their ecological validity^14,21–23^.

It is now technologically feasible to conduct quantitative mobility assessments during real-world walking, defined as unsupervised, unscripted walking behavior that occurs in non-simulated everyday situations^14,24,25^. Walking-related digital mobility outcomes (DMOs) including gait speed, step length, and step count are increasingly used to quantify gait quality in multiple medical conditions. Emerging evidence suggests that DMOs may be sensitive, ecologically valid markers of health status^14,21,23^, but they are unvalidated and therefore not yet accepted as mainstream research and clinical assessment tools. This gap has sparked multidisciplinary calls to validate and qualify (i.e., seek regulatory approval for) DMOs as clinical endpoint measures^26–31^. These calls suggest that collaboration across traditional clinical divides will accelerate the qualification process, which entails patient engagement, extensive technical validation, large clinical studies, and an intensive review by regulatory authorities^12,28,29,31^. This process must prove that DMOs are technically feasible to measure, relevant to patients, clinically meaningful, and cost-effective, among other considerations. In this context, clinical meaningfulness is judged by three psychometric properties: construct validity (i.e., they measure what they are supposed to measure), predictive validity (they are associated with important clinical outcomes such as mortality), and responsiveness (they change in response to effective interventions)^32–34^.

Many DMOs have been investigated and proposed, but systematic evidence on their psychometric properties is often lacking. This is in part due to fragmentation of the literature by discipline, terminology, and methodology—both within and between clinical disciplines. Systematic evidence is beginning to accumulate, but is generally limited to clinical settings and specific medical conditions, DMOs, or psychometric properties^3,35–37^. These reviews, although foundational, provide a narrow and incomplete understanding of the research landscape. An overarching view of existing evidence is needed to guide strategic priority setting, inform the design of validation efforts, and identify common research goals—and therefore opportunities for collaboration—which exist across traditional research domains.

### Objective

**Fig. 1:**
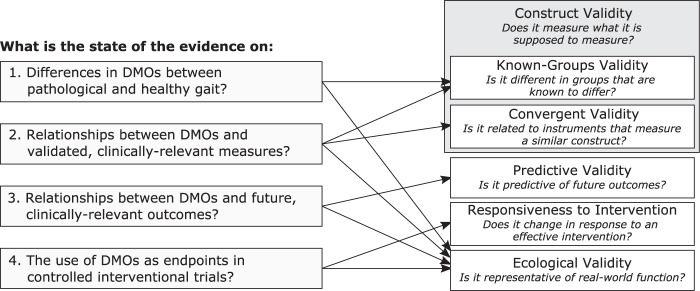
Research questions (left) and psychometric properties (right) addressed by this review. DMO digital mobility outcome.

The aim of this scoping review is to generate cross-disciplinary maps of existing evidence on the clinical meaningfulness of DMOs. We stratified our review by four research questions (Fig. 1) designed to map evidence pertaining to the known-groups validity, convergent validity, predictive validity, responsiveness, and ecological validity of a predefined set of DMOs. The resulting maps identify commonalities across disciplinary divides, suggest promising DMOs for further validation, and outline current research gaps. Although walking impairment is of interest in many medical conditions, it was impossible to map the entire research field in a single review. We selected four diverse medical conditions as exemplars, representing diverse etiologies and patterns of mobility impairment: Parkinson’s disease (PD), multiple sclerosis (MS), chronic obstructive pulmonary disease (COPD), and proximal femoral fracture (PFF)^31^. They were selected due to their prevalence, impact on quality of life, economic burden, and evidence base^35,37–39^. Walking impairment is known to play a central role in the patient experience of each of these conditions^12,40–44^. These conditions are the focus of Mobilise-D, an Innovative Medicines Initiative 2 Joint Undertaking that aims to develop and validate DMOs for regulatory and clinical endorsement^45^.

## Results

### Characteristics of included studies

**Fig. 2:**
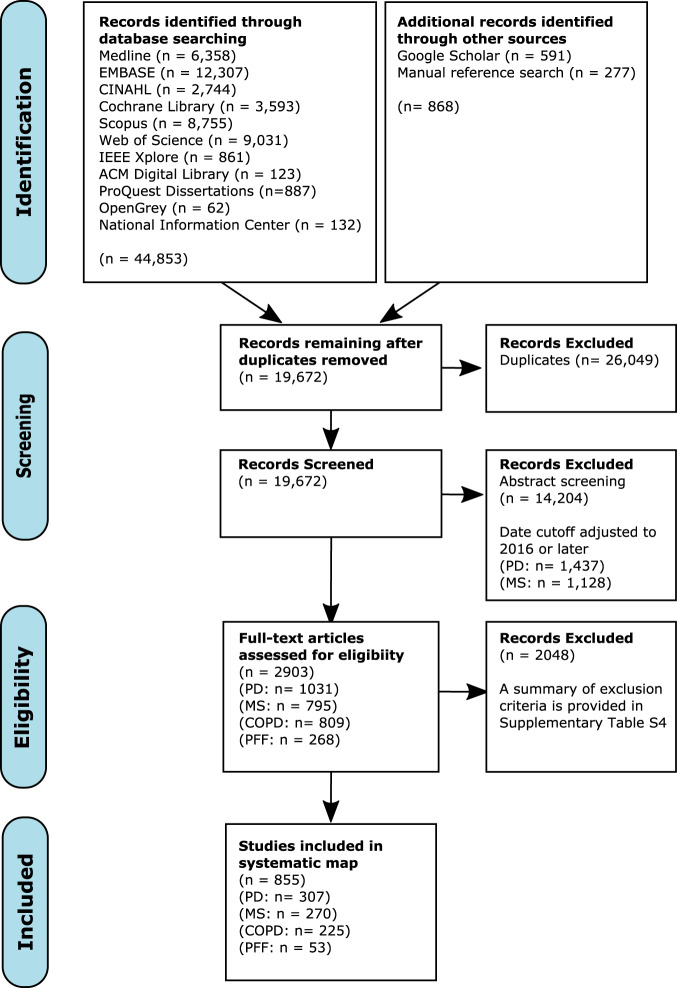
PRISMA flow diagram. This diagram shows how records were screened for eligibility in this review.

Searches yielded 19,672 unique records, of which 2903 were deemed eligible for full-text review. Of these, 855 records were eligible for inclusion (PD: *n* = 307; MS: *n* = 270; COPD: *n* = 225; PFF: *n* = 53), representing 5019 unique analyses from 775 studies (Fig. 2). The list of included records is available on our project repository^46^. Reviewer agreement was substantial at the abstract (16 raters, weighted Cohen’s *κ* = 0.77, Fleiss’ *κ* = 0.56) and full-text stages (22 raters, weighted Cohen’s *κ* = 0.75). Gait speed was studied most frequently in all medical conditions except COPD, which favored daily step count (Supplementary Fig. 1). Characteristics of included studies and their populations are provided in Supplementary Table 8. Most studies were small (median [IQR]: 50 [30–94] participants) and included populations with moderate median disease severity. We observed substantial methodological heterogeneity both within and between the medical conditions, although the methods were often unclearly reported (Table 1).

**Table 1:** Walking conditions and measurement methods used in included studies.

	PD, *n* = 265	MS, *n* = 250	COPD, *n* = 193	PFF, *n* = 48
Measurement method				
Stopwatch	49 (18.5%)	158 (63.2%)	37 (19.2%)	34 (70.8%)
Video/optoelectronic system	65 (24.5%)	24 (9.6%)	4 (2.1%)	0 (0.0%)
Instrumented walkway	51 (19.2%)	34 (13.6%)	10 (5.2%)	8 (16.7%)
Instrumented treadmill	10 (3.8%)	6 (2.4%)	2 (1.0%)	0 (0.0%)
Instrumented environment	2 (0.8%)	1 (0.4%)	0 (0.0%)	0 (0.0%)
Wearable sensor^a^ (hip/waist)	32 (12.1%)	16 (6.4%)	49 (25.4%)	0 (0.0%)
Wearable sensor (other/mixed locations)	58 (21.9%)	31 (12.4%)	74 (38.3%)	4 (8.3%)
Pedometer	1 (0.4%)	0 (0.0%)	31 (16.1%)	1 (2.1%)
Mobile phone	1 (0.4%)	1 (0.4%)	0 (0.0%)	0 (0.0%)
Video gaming system (e.g., Kinect)	6 (2.3%)	4 (1.6%)	0 (0.0%)	0 (0.0%)
Other	14 (5.3%)	3 (1.2%)	4 (2.1%)	4 (8.3%)
Measurement setting				
Clinic/lab	252 (95.1%)	240 (96.0%)	63 (32.6%)	41 (85.4%)
Home/real world	20 (7.5%)	25 (10.0%)	135 (69.9%)	5 (10.4%)
Walking bout length				
Short walk (≤1 min or <20 m)	204 (77.0%)	211 (84.4%)	35 (18.1%)	34 (70.8%)
Longer walk (>1 min or 20 m)	49 (18.5%)	54 (21.6%)	21 (10.9%)	13 (27.1%)
Real-world walking bouts	17 (6.4%)	24 (9.6%)	139 (72.0%)	4 (8.3%)
Unclear	13 (4.9%)	10 (4.0%)	3 (1.6%)	0 (0.0%)
Walking bout speed				
Habitual speed	205 (77.4%)	95 (38.0%)	35 (18.1%)	23 (47.9%)
Fast speed	34 (12.8%)	158 (63.2%)	22 (11.4%)	19 (39.6%)
Set speed (i.e., on a fixed-speed treadmill)	10 (3.8%)	5 (2.0%)	2 (1.0%)	0 (0.0%)
Averaged bouts of variable speeds	3 (1.1%)	0 (0.0%)	0 (0.0%)	0 (0.0%)
Real-world walking bouts	17 (6.4%)	23 (9.2%)	140 (72.5%)	3 (6.2%)
Unclear	32 (12.1%)	19 (7.6%)	6 (3.1%)	8 (16.7%)

Data are presented as *n* (%) of included studies. Multiple records were identified for several studies; thus, the total number of studies differs from the total number of records. The sum of percentages may exceed 100%, as studies often reported results for multiple measurement methods or walking conditions.

Measurement method, measurement setting, walking bout length, and walking bout speed indicate the categories of walking conditions reported in included studies.

*PD* Parkinson’s disease, *MS* multiple sclerosis, *COPD* chronic obstructive pulmonary disease, *PFF* proximal femoral fracture.

^a^Wearable sensors refer to any wearable data acquisition device other than pedometers, including accelerometers and inertial measurement units.

### Known-groups validity

**Fig. 3:**
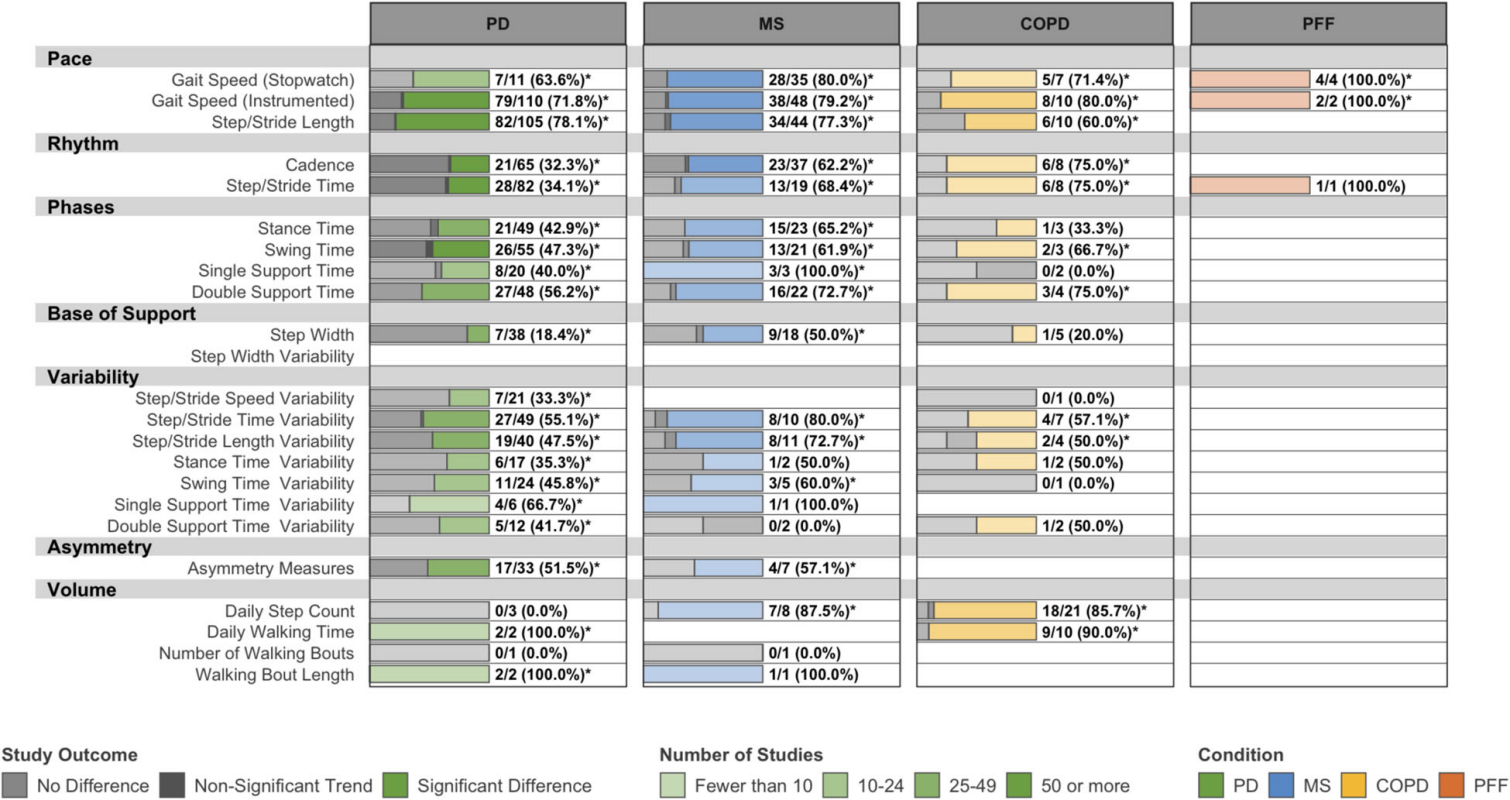
Known-groups validity: number and outcome of eligible studies assessing differences in DMOs between patients and healthy controls. PD Parkinson’s disease, MS multiple sclerosis, COPD chronic obstructive pulmonary disease, PFF proximal femoral fracture. Data are presented as: Number of studies with statistically significant differences between groups/Total studies (%). DMOs known to be highly intercorrelated were grouped (i.e., step length and stride length), and all DMOs were organized according to previously established domains of gait. *Proportion of studies exceeds the expected false-positive rate as determined by Bernoulli hypothesis testing and Benjamini–Hochberg adjustment.

Overall, 282 studies investigated differences in DMOs between healthy and pathological gait (Fig. 3) and 137 studies compared DMOs across disease severity strata (Supplementary Fig. 4). Several DMOs exhibited consistent evidence of known-groups validity in PD, MS, and COPD, although few investigated differences between known groups for any DMO in PFF. Gait speed, step/stride length, step/stride length variability, and measures describing the support phase of gait were consistently different between known groups, although the evidence is limited for disease severity strata in COPD. DMOs describing cadence, step/stride time, and daily step count were consistently different between known groups in MS and COPD, but less so in PD.

### Convergent validity

**Fig. 4:**
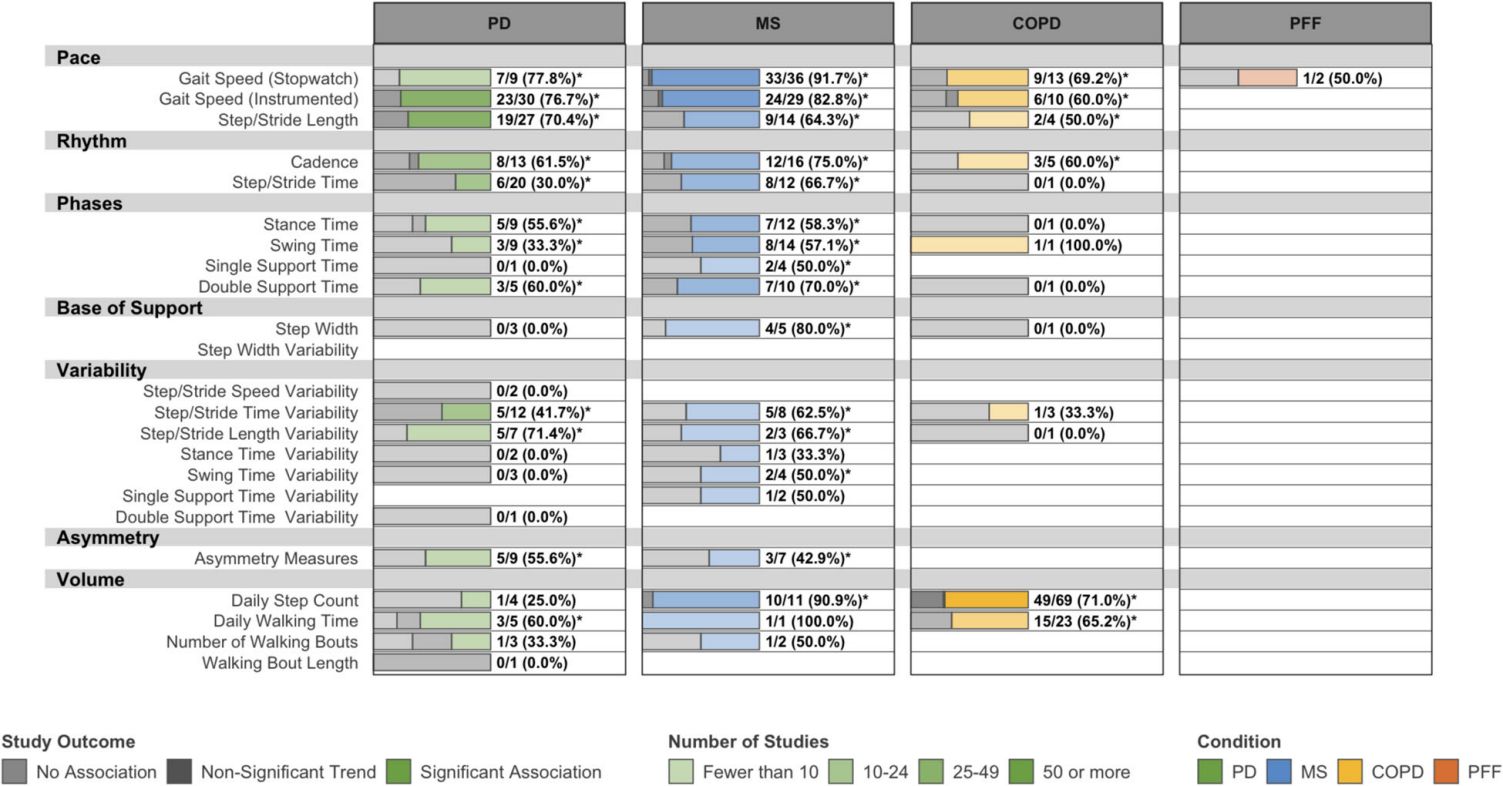
Convergent validity: associations between DMOs and disease severity measures. PD Parkinson’s disease, MS multiple sclerosis, COPD: chronic obstructive pulmonary disease, PFF: proximal femoral fracture. Data are presented as: Number of studies with statistically significant associations between DMOs and measures of disease severity/Total studies (%). Disease severity measures include the UPDRS, UPDRS-III, and Hoehn & Yahr scale in PD, EDSS, and PDDS in MS, FEV_1_ % predicted and GOLD stage in COPD, and patient- or physician-rated global measures of improvement in all four conditions. Most relevant measures in PFF fell under different categories, such as activities of daily living. DMOs known to be highly intercorrelated were grouped (i.e., step length and stride length), and all DMOs were organized according to previously established domains of gait. *Proportion of studies exceeds the expected false-positive rate as determined by Bernoulli hypothesis testing and Benjamini–Hochberg adjustment.

We identified 378 studies that investigated associations between DMOs and validated measures of condition severity, lower-extremity function, health-related quality of life, and other constructs. Gait speed, step/stride length, cadence, and step/stride time exhibited consistent relationships with measures of condition severity (Fig. 4) and lower-extremity function (Supplementary Fig. 5). Mapped associations between DMOs and measures of balance, falls, and health-related quality of life are provided in Supplementary Figs. 6–9. Gait speed, daily step count, and daily walking time were consistently associated with health-related quality of life in all conditions. Gait speed, step/stride length variability, and step/stride time variability were most consistently related to balance and falls, although this primarily reflected studies in PD and MS.

### Predictive validity

Only 33 studies investigated the predictive validity of DMOs (PD: *n* = 10; MS: *n* = 7; COPD: *n* = 14; PFF: *n* = 2). Most studies (22 of 33, 66.7%) adjusted at least some of their analyses for known predictors or common confounders. In PD, several DMOs were related to future disease progression, falls, physical function, and cognition^47–58^ (Supplementary Fig. 10) In MS, gait speed was associated with disease progression^59,60^, future falls^61,62^, and functional status^63^. One study identified a relationship between daily step count and disease progression^64^. Another identified a relationship between stride time variability, but not stride speed variability, and falls^65^. In COPD, gait speed^66–70^ and daily step count^71–75^ demonstrated relationships with mortality. One study identified a relationship between step count and disease progression^72^. Evidence for relationships with exacerbations^76,77^, activities of daily living^72^, health-related quality of life^72^, and healthcare utilization^69,74,78^ was limited or inconsistent. In PFF, one study found a relationship between gait speed and healthcare utilization^79^, while the relationship between gait speed and activities of daily living was inconsistent^79,80^.

### Responsiveness to intervention

**Fig. 5:**
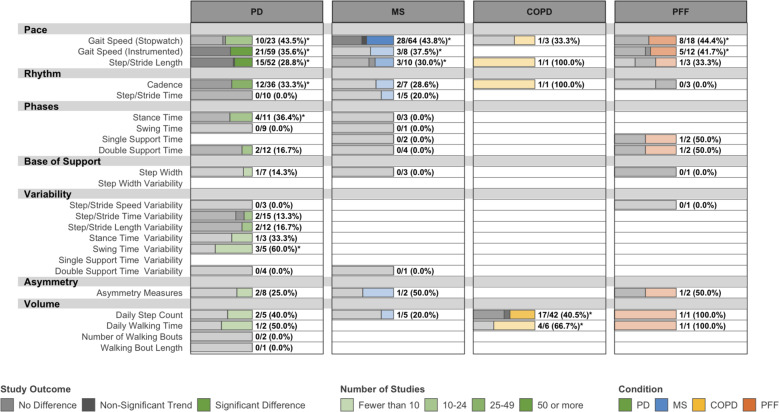
Responsiveness of DMOs used as primary or secondary endpoints in all eligible interventional studies. PD Parkinson’s disease, MS multiple sclerosis, COPD chronic obstructive pulmonary disease, PFF proximal femoral fracture. Data are presented as: Number of studies with statistically significant differences between groups/Total studies (%). Interventions in eligible studies were not necessarily effective, and this map may underestimate the responsiveness of DMOs. DMOs known to be highly intercorrelated were grouped (i.e., step length and stride length), and all DMOs were organized according to previously established domains of gait. *Proportion of studies exceeds the expected false positive rate as determined by Bernoulli hypothesis testing and Benjamini–Hochberg adjustment.

**Fig. 6:**
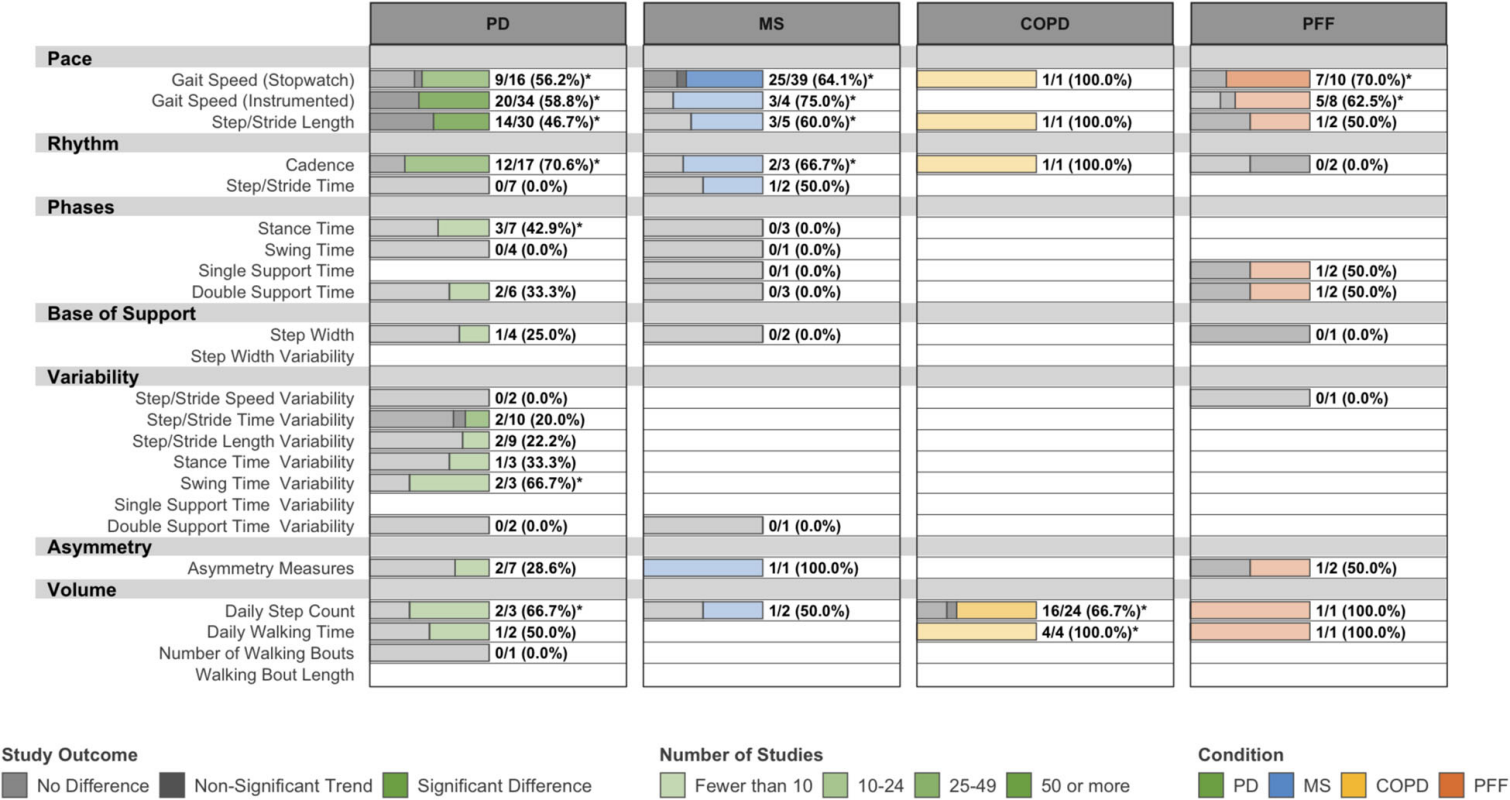
Responsiveness of DMOs used as primary or secondary endpoints when a studied intervention was effective. PD Parkinson’s disease, MS multiple Sclerosis, COPD chronic obstructive pulmonary disease, PFF proximal femoral fracture. Data are presented as: Number of studies with statistically significant differences between groups/Total studies (%). This map may overestimate the responsiveness of DMOs, which were occasionally used as sole primary outcomes (i.e., gait speed and step count), since negative results could be due either to the DMO’s responsiveness or to the intervention’s efficacy. DMOs known to be highly intercorrelated were grouped (i.e., step length and stride length), and all DMOs were organized according to previously established domains of gait. *Proportion of studies exceeds the expected false-positive rate as determined by Bernoulli hypothesis testing and Benjamini–Hochberg adjustment.

We identified 208 studies that used DMOs as outcome measures in controlled interventional trials. Of these, 140 (67.3%) reported using a DMO as a primary outcome and 79 (38.0%) reported using a DMO as a secondary outcome. However, many studies reported several “primary” outcomes and it was often unclear which outcomes, if any, were used in the power analysis. Studies were generally designed to evaluate the efficacy of interventions rather than the responsiveness of DMOs; therefore, evidence of DMOs’ responsiveness could not be clearly disentangled from the efficacy of the various experimental interventions. Thus, we created two maps to estimate responsiveness. Figures 5 and 6 map analyses from all included studies and studies in which interventions were “effective,” respectively. For our purposes, “effective” interventions are those which yield significant differences in any primary endpoint between experimental and control or comparator arms. The former is likely to underestimate responsiveness (it is confounded by the true efficacy of the experimental interventions), and the latter is likely to overestimate it (it is biased in favor of successful studies that used DMOs as primary outcomes). Gait speed, step/stride length, cadence, daily step count, and walking time often responded to “effective” interventions in all conditions, although outcomes were relatively inconsistent.

### Ecological validity

**Fig. 7:**
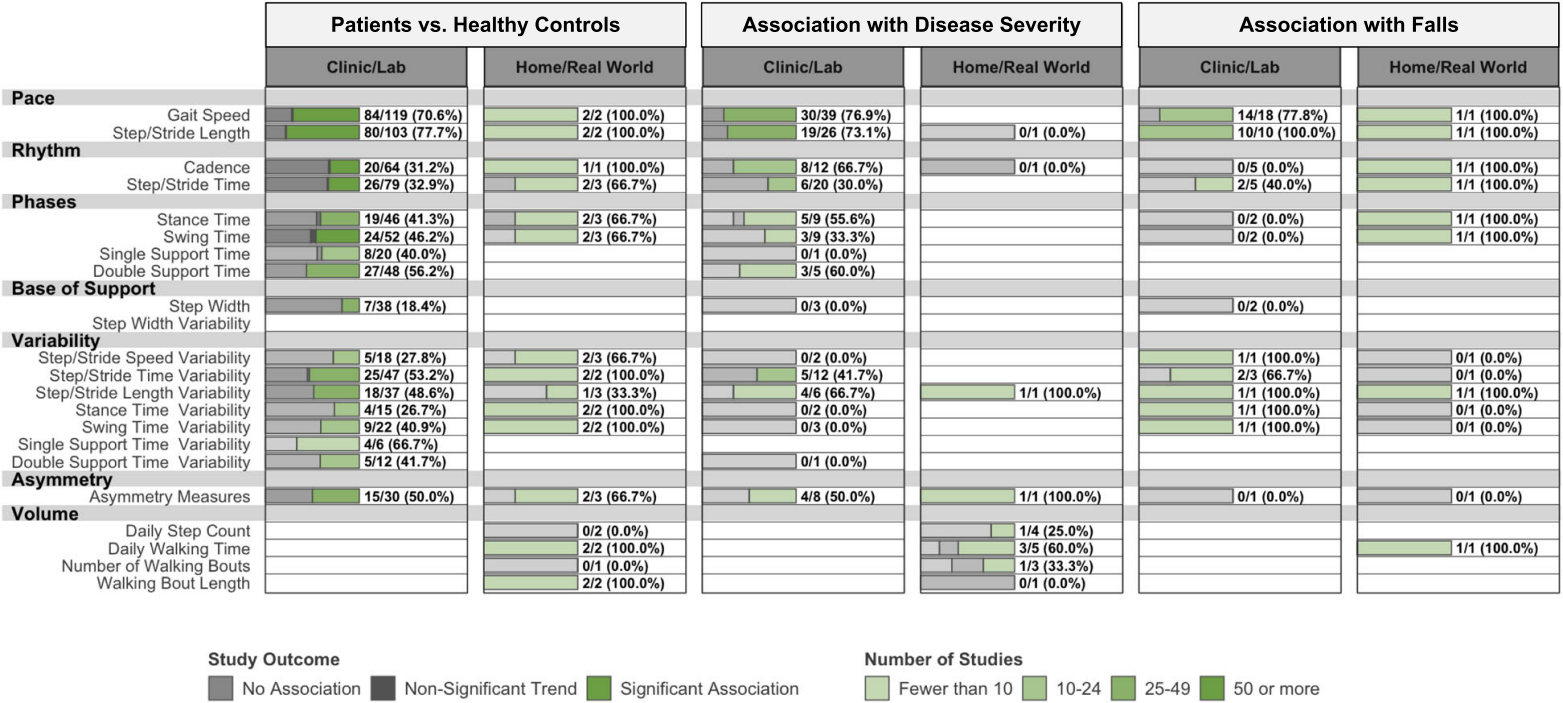
Ecological validity of DMOs in Parkinson’s disease: DMOs collected in clinical vs real-world environments. Data are presented as: Number of studies with statistically significant associations between DMOs and measures of lower-extremity function/Total studies (%). DMOs known to be highly intercorrelated were grouped (i.e., step length and stride length), and all DMOs were organized according to previously established domains of gait. *Proportion of studies exceeds the expected false-positive rate as determined by Bernoulli hypothesis testing and Benjamini–Hochberg adjustment.

Excluding measures of daily walking volume, only 17 studies measured spatiotemporal DMOs during real-world walking (PD: *n* = 8; MS: *n* = 5; COPD: *n* = 2; PFF: *n* = 2). Relationships exhibited by real-world and in-clinic DMOs are compared for PD^81–88^ and MS^21,89–92^ in Fig. 7 and Supplementary Fig. 12, respectively. In COPD, real-world walking cadence differed from healthy controls^93^ and real-world gait speed was associated with disease severity^94^. In PFF, timed gait speed tests were conducted at home in two studies^95,96^. These tests were not responsive to intervention, although interventions in both studies were found to be ineffective. These relationships were similar to those observed in clinical settings, but such comparisons were qualitative.

### Assessment of bias

Manual inspection revealed key differences in research strategy between the medical conditions. Records in PD were more likely to study specific subpopulations with gait impairments (e.g., fallers, individuals with freezing of gait). Several records in MS and PD, but not COPD or PFF, specifically studied populations with early-stage disease. The body of literature on PD, COPD, and PFF appeared to exhibit a survivorship bias (in this case, the tendency for healthier-than-average individuals with a given characteristic to be included in a study) with respect to age and condition severity. Only studies in MS, which represented a younger population, reflected the full range of disease severity and demonstrated the expected colinearity of age and disease severity (Supplementary Fig. 2). This is likely due to an association between age, condition severity, and comorbidities or cognitive impairment, which were often exclusion criteria in included studies.

Meta-regression showed that conference abstracts (adjusted odds ratio [95% confidence interval]: 2.44 [1.59–3.76], *p* < 0.001), studies with fast walking assessments (1.54 [1.10–2.17], *p* = 0.02), and studies on at-risk subgroups such as fallers (2.03 [1.47–2.80], *p* < 0.001) were more likely to report significant results than their counterparts. Conversely, studies on populations with mild disease severity (0.46 [0.34–0.61], *p* < 0.001) were less likely to report significant findings than those with moderate severity. In studies comparing pathological to healthy gait, those that matched patients and controls for gait speed were less likely to report significant findings for any DMO (0.39 [0.18–0.83], *p* = 0.014). Contrary to our expectations, adjusted models were more likely to yield significant findings than univariate analyses in studies investigating the prognostic value of DMOs. This suggests that DMOs that did not reach significance in multivariate models were not consistently reported and that our maps may overestimate the true repeatability of these relationships. No other study characteristics were associated with study outcomes. Sensitivity analyses yielded similar estimates of all effects. It is important to note that these relationships are observational, and may not hold at the individual or study level^97^. They merely suggest that methodological and population heterogeneity contributed to the inconsistencies observed in our maps. Detailed results of these analyses are provided in the Supplementary materials (Supplementary Notes 3 and 5, Supplementary Fig. 3, and Supplementary Table 7).

### Qualitative appraisal of existing evidence

We identified several notable evidence gaps. Few records studied the predictive validity of DMOs in any of the four conditions. Only gait speed and step count were regularly used as outcomes in interventional studies. Few DMOs were studied regularly in COPD and PFF.

Despite these gaps, evidence consistently supported the validity of gait speed, step/stride length, cadence, step/stride time, step/stride time variability, and daily step count whenever it was available (Table 2). In PD, more positive evidence was available for daily walking time than step count, but evidence supporting these measures was similar in the other conditions. Several DMOs exhibited evidence in PD and MS but lacked evidence in the other two conditions. Additional detail is provided in Supplementary Tables 10–13.

**Table 2:** Qualitative appraisal of existing evidence.

Gait domain	Digital mobility outcome	PD	MS	COPD	PFF
Pace	Gait speed	++	++	++	++
	Step/stride length	++	+	+	?
Rhythm	Cadence	++	++	+	?
	Step/stride time	+	+	?	?
Phase	Stance time	++	+	?	?
	Swing time	+	+	?	?
	Single support time	+	+	?	?
	Double support time	+	+	?	?
Base of support	Step width	−	+	?	?
	Step width variability	−	−	?	?
Variability	Step/stride speed variability	+	?	?	?
	Step/stride length variability	++	+	?	?
	Step/stride time variability	+	+	?	?
	Stance time variability	+	?	?	?
	Swing time variability	+	+	?	?
	Single support time variability	?	+	?	?
	Double support time variability	?	−	?	?
Asymmetry	All asymmetry measures	++	+	?	?
Volume	Daily step count	+	+	++	?
	Daily walking time	+	?	+	?
	Number of walking bouts	−	?	?	?
	Walking bout length	?	+	?	?

*PD* Parkinson’s disease, *MS* multiple sclerosis, *COPD* chronic obstructive pulmonary disease, *PFF* proximal femoral fracture.

## Discussion

Recent calls to validate real-world DMOs are based on three premises: that DMOs are clinically meaningful, that relationships observed in clinical settings translate to real-world walking, and that opportunities for collaboration across clinical disciplines exist^30,31^. This review conditionally supports these premises. Mobility indeed appears to be a concept of cross-disciplinary clinical interest. Multiple DMOs were regularly studied in the four included conditions and consistently exhibited evidence of construct validity, predictive validity, and responsiveness. Few studies measured real-world walking in this review, but those that did provide provisional evidence that relationships observed in clinical settings translate to real-world walking.

While condition- and context-specific validation studies are certainly required for the formal validation of DMOs^29,98^, it appears that collaborative approaches to validation can speed this process^30,31^. The regulatory pathway for validating and qualifying DMOs—and digital outcomes in general—is taking shape due to the collaborative efforts of regulators, industry, academics, and precompetitive consortia^28,29,98–102^. The time is right for collaborative development of terminology, algorithms, methods, and evaluation frameworks for mutually interesting DMOs, which may streamline the validation of DMOs in PD, MS, COPD, PFF, and other medical conditions.

However, the volume of existing evidence varied across conditions and DMOs. Compared to PD and MS, evidence in PFF and COPD was sparse and concentrated on fewer DMOs. These differences, plus the differences in prevailing methodologies, suggest disparate research strategies between the conditions. DMOs appear to be more established in some conditions than others. Even in recent studies, uninstrumented gait speed tests were used more frequently than other spatiotemporal DMOs in MS, COPD, and PFF. These tests are widely used, well-established, inexpensive, and simple to implement. However, they are limited by sensitivity to methodology, Hawthorne effects, and other shortcomings^103,104^. Current methods to measure other DMOs are newer, less mature, and more expensive, requiring significant infrastructure and technical expertise^105^. As these factors likely influence the adoption and study of DMOs, we do not consider lack of evidence to constitute negative evidence or inferiority in this review. These gaps are merely areas in which more evidence must be established.

The purpose of scoping reviews is to map research fields and set an agenda for future research^106,107^. The relationships observed here provide clues on the contexts in which DMOs might be useful as outcome measures and suggest gaps that should be addressed to inform DMO validation.

### Predictive Validity

Endpoint qualification requires evidence that DMOs are associated with “hard” clinical outcomes such as falls, hospitalization, and mortality. Relationships between these outcomes and in-clinic gait speed are established in many conditions^108,109^, including those studied here. However, with few exceptions, evidence on the predictive validity of other DMOs is sparse. Despite the purported potential of spatiotemporal parameters and real-world DMOs as clinical measures, further work is needed to confirm their predictive validity before they can be considered for regulatory qualification.

### Responsiveness to Intervention

This review identified preliminary evidence for the responsiveness of common DMOs. However, included studies were not specifically designed to assess the responsiveness of DMOs; they were designed to test the efficacy of interventions. It is not yet clear which DMOs are responsive to which types of interventions, nor is it clear what constitutes clinically meaningful changes in these DMOs. The context-dependency and relative magnitude of DMOs’ responsiveness should be confirmed against “gold standard” outcomes through adequately powered interventional studies and meta-analyses. Any future work should report measures of effect size to quantify the responsiveness of DMOs.

### Ecological validity

Scripted walking assessments, which test functional capacity at a single timepoint, are not necessarily representative of habitual or spontaneous walking behavior^21,110,111^. If DMOs are to be used as real-world measures or interpreted as “ecologically valid,” the psychometric properties of DMOs measured during real-world walking must be established. At the time of our search, records measuring real-world walking were relatively rare. Real-world walking assessment remains technically and logistically challenging^105^. The performance of existing algorithms, which are usually validated under controlled clinical conditions, varies with changing environment, activities, and walking speed^112^. The effect of this variation on DMOs’ clinical utility is unclear. While research on real-world walking has recently accelerated in PD and MS, future work should enrich this evidence. As a priority, this work should supplement the pioneering studies that conduct head-to-head comparisons of DMOs measured in the clinic and in the real world^84,110,111^. In the near future, literature on real-world DMOs should be systematically reviewed to establish similarities and differences between real-world and in-clinic walking assessments.

### Importance to patients

The clinical perspectives and psychometric properties discussed here, while necessary, are ultimately insufficient to guide DMO selection and validation. This review mapped hundreds of promising relationships from studies conducted in various contexts, populations, and settings, begging the question, “Of the DMOs and relationships we *can* validate, which *should we* validate?”

Regulatory bodies such as the Food and Drug Administration, the European Medicines Agency, and local Health Technology Assessment bodies expect this question to be addressed from the patient perspective^113–115^. The onus is on researchers to prove that new digital outcomes are important and meaningful within the context of patients’ daily lives^114,116^. This refers not only to the construct the measure assesses but also to the level of change the measure can detect. However, relationships between DMOs and the constructs that matter to patients are not always direct. While some DMOs (i.e., gait speed, daily step count) are readily interpretable, others may have little intuitive or practical meaning to anyone other than a gait specialist (i.e, stance time variability). The relationships between DMOs and meaningful constructs must be established both statistically and through early patient engagement, journey mapping, and formal qualitative research, and collaborative agenda setting. Existing guidance^113,115,117,118^ and worked examples from past projects^12,102^ can shape this interaction. The maps generated in this study can be used to match candidate DMOs with prioritized walking-related constructs and experiences. These candidate DMOs should then be considered specifically when addressing the evidence gaps described here.

### Generalizability and context

Relationships between DMOs, condition severity, and physical function in all four conditions suggest that DMOs may be useful to monitor disease progression or changes in mobility status over time. Similarly, cross-sectional and longitudinal associations between DMOs and falls suggest that DMOs may be useful to quantify fall risk in PD and MS. However, the maps presented here should be treated as directional. The included conditions are highly heterogeneous, representing an array of symptoms under single diagnostic umbrellas. It is entirely possible that the utility of any given DMO is context-dependent, differing between environments, early and late-stage disease, during an acute health event or exacerbation, or between disease subtypes. Examples include individuals with relapsing vs. progressive courses^119^ or ataxic vs. paretic gait^120^ in MS, freezing of gait^121^, orthostatic hypotension^122^, tremor-dominant vs. postural instability gait disorder subtypes^123^ in PD, oxygen users in COPD^124^, or those with different fracture and surgery types in PFF^125^. For many subpopulations, additional original research may be required. When evidence exists, nuanced perspectives on DMOs’ clinical utility can be explored through a systematic review.

### Strengths and limitations

Our maps aggregated a large, heterogeneous body of literature to identify overarching trends, inform future research, and identify opportunities for cross-disciplinary collaboration. Technical and clinical subject matter experts took part in multidisciplinary review teams, guiding the design of the review and interpretation of the results. Despite its rigor, this review has several notable limitations. For feasibility, we limited included records on PD and MS to those published in 2016 or later. Thus, findings for these two diseases should be interpreted as trends in the literature, rather than an exhaustive tabulation of existing evidence. However, methods remained systematic and data saturation was generally observed. Inconsistent reporting necessitated the use of a relationship’s statistical significance, rather than its effect size, in our maps and analyses. Therefore, trends should be interpreted as the repeatability, rather than strength, of observed relationships. Additional systematic reviews and meta-analyses are needed to estimate the strength of key relationships and assess the quality of existing evidence. Despite the breadth of mobility symptoms and disease trajectories covered in the four included medical conditions, the relationships observed here may differ in other conditions, or even within specific subpopulations of the included conditions. Although many DMOs were included, evidence is emerging for other DMOs such as sample entropy, Lyapunov exponents and detrended fluctuation analysis, which are of special interest in real-world assessments^126–131^. As these measures mature, this review should be updated and expanded to include additional DMOs. Finally, we present our results at a high level and many interesting subanalyses were not conducted. We hope that this work will inspire and enable a deeper investigation into the topics discussed here.

### Conclusions

Existing evidence supports cross-disciplinary validation efforts for gait speed, step and stride length, cadence, and step count, but is inconsistent or lacking for other DMOs. The relationships exhibited by these DMOs were similar across conditions, signaling potential opportunities for cross-disciplinary collaboration. Future work should include further epidemiological studies, systematic reviews, and meta-analyses to confirm and quantify the relationships observed in this scoping review.

## Methods

### Review methodology

We followed the scoping review framework developed by Arksey and O’Malley and advanced by Levac et al.^106,107^. This framework consists of six stages: (1) identifying the research question, (2) identifying relevant studies, (3) selecting studies, (4) charting the data, (5) collating, summarizing, and reporting results, and (6) consulting with relevant stakeholders. Study conduct and reporting adhered to the PRISMA-ScR (PRISMA Extension for Scoping Reviews) guidelines for scoping reviews^132^. A detailed review protocol was designed and published a priori^133^, and is summarized here.

### Identifying relevant studies

Search strategies were iteratively developed and tested in exchange with a research librarian and subject matter experts. In November 2019, the librarian searched 11 databases for scientific and gray literature (MEDLINE, EMBASE, CINAHL, Cochrane Library, Scopus, Web of Science, IEEE Xplore, ACM Digital Library, ProQuest Dissertations, OpenGrey, National Information Center’s Projects in Progress Database). Final searches with structure (mobility terms) AND (population terms) identified all English-language abstracts published between January 1999 and November 2019. Similar searches in Google Scholar and manual collation of references supplemented this corpus. The search strategy for MEDLINE is provided in Supplementary Table 1 and all search strategies are provided on the project repository^46^.

### Selecting studies and charting the data

All relevant definitions, eligibility criteria, reference sheets, and data extraction forms are provided in Supplementary Note 1, Supplementary Table 2, or the project protocol^133^. To be eligible, a record must have reported an original analysis that addressed at least one of our research questions with respect to an included DMO in an included population. For the sake of feasibility, we prespecified a list of included DMOs (Supplementary Table 3), limited assessments of construct validity and predictive validity to predefined lists of validated measures, and set a lower limit of ten patients per analysis (or study arm, in the case of interventional trials). We did not otherwise exclude based on methodology. Predefined lists were developed by internal panels of clinical, technical, and research experts. Texts published in any language spoken within our research group (English, German, Spanish, French, Italian, Portuguese, Danish, Norwegian, Swedish, Hebrew, Dutch, Catalan, Russian) were eligible.

We assessed eligibility through abstract and full-text screening. All reviewers were trained, piloted study materials, and completed consistency checks before each screening phase. Records were included in full-text screening if a single reviewer deemed an abstract eligible, while rejection by two reviewers was required to exclude. Full-text screening was conducted by those with relevant clinical and technical expertise. One reviewer screened each full-text and, if eligible, extracted data. One of three senior reviewers (A.P., N.C., and H.G.) then checked each review for accuracy. Disagreements were resolved through discussion or, when necessary, a third review. Records stemming from the same study were identified through keyword and author searches and confirmed via manual review. These records were linked and duplicate analyses were removed. The net agreement was assessed via Fleiss’ *κ*^134^ and individual agreement between each reviewer and the primary reviewer (A.P.) was monitored via Cohen’s *κ*^135^. Record screening and data management were conducted in DistillerSR (Evidence Partners, Ottawa, Canada).

Scoping reviews map broad, previously uncharted bodies of literature; thus, Arksey and O’Malley’s framework allows for the reflexive adaptation of eligibility criteria to ensure scope remains manageable^106^. We made three such adaptations according to a predefined process. First, we added a second, condition-specific abstract screening phase because limited disease-area knowledge led to the overinclusion of ineligible records. Exclusion during this phase was restricted to criteria associated with disease-specific knowledge. Due to the volume of relevant literature, we limited full-text review in PD and MS to literature published during or after 2016. Therefore, maps of PD and MS must be interpreted as trends in recent research, rather than an exhaustive tabulation of evidence. These maps were monitored for data saturation, defined as “the point in the research process when no new information is discovered in data analysis… [and the] researcher can be reasonably assured that further data collection would yield similar results.”^136^ Maps of COPD and PFF remained exhaustive. Finally, we shifted from a parallel full-text review paradigm, in which all records are reviewed independently in duplicate, to the review/quality-control paradigm described above. All changes were made between the abstract and full-text review stages, approved by the study team, and applied to all records.

### Collating, summarizing, and reporting results

We systematically mapped the results of eligible records through frequency analysis, which was stratified by medical condition and DMO. Each map reflects the volume and outcome of existing evidence on a specific association, relationship, or characteristic of DMOs, as described in Table 3. For our purposes, “volume” of evidence refers to the number of unique analyses reported in the corpus, and “outcome” refers to the proportion of these analyses which yielded statistically significant results according to the authors’ original analyses. It is important to note that this approach describes an association’s repeatability, rather than its strength. The latter would be more appropriately described by measures of effect size, but because effect sizes were not consistently reported or interpreted in included studies, they were not feasible to map. This issue should be the focus of future systematic reviews and meta-analyses.

**Table 3:** Psychometric properties mapped in this review.

Property	Maps generated in this review
Known-groups validity	Number and proportion of analyses per DMO and medical condition, which found a statistically significant difference (1) between pathological and healthy gait, or (2) between disease severity strata
Convergent validity	Number and proportion of analyses per DMO and medical condition, which found a statistically significant, cross-sectional association between a DMO and validated measures of relevant constructs (e.g., disease severity, physical function, health-related quality of life, etc.)
Predictive validity	Number and proportion of analyses per DMO and medical condition, which found a statistically significant association between a DMO measured at baseline and a clinically relevant outcome at follow-up (i.e., mortality, physical function, healthcare utilization, etc.)
Responsiveness to intervention	Number and proportion of analyses per DMO and medical condition, which found a significant difference between experimental and control groups in an interventional study
Ecological validity	DMOs measured in clinical and real-world settings were mapped separately and trends were compared qualitatively

For ease of interpretation, DMOs were organized into the previously established domains Pace, Rhythm, Phase, Base of Support, Variability, and Symmetry^137–141^. DMOs within each of these domains are known to exhibit similar characteristics and are highly inter-correlated. Step count, walking time, walking bout length, or duration were categorized as “Volume of Walking.” Operational definitions of these domains and their associated DMOs are provided in Supplementary Table 3.

### Assessing risk of bias

Critical appraisal of individual studies is not required for scoping reviews^132^, and was not conducted here. However, given the heterogeneity of included records, it was necessary to identify sources of bias and assess sensitivity to study design before we could confidently interpret our maps.

Because our data were comprised of statistical tests reported by the authors themselves, it was important to consider the potential influence of statistical heterogeneity, multiple testing, and false positives. Inclusion of underpowered analyses or those unadjusted for multiple testing could inflate the observed repeatability of mapped relationships. It was, therefore, necessary to confirm whether observed proportions exceeded type 1 error rates (i.e., false positives) expected under conservative conventional assumptions. Observed distributions were subjected to single-population Bernoulli hypothesis tests with a mean of zero and an expected false-positive rate of 5% (assuming the conventional *α* = 0.05). These hypothesis tests were then adjusted for multiple testing through a Benjamini–Hochberg procedure^142^. When the proportion of studies reporting statistically significant results exceeded expected false-positive rates, this was indicated on the maps. The proportions themselves were not adjusted and should be interpreted with the potential impact of statistical heterogeneity in mind.

We also assessed clinically plausible sources of bias and effect modification in the entire corpus through manual inspection and random-effects meta-regression^97,143,144^. Potential effect modifiers included the speed and length of walking bouts, statistical analysis methods, and the size, median age, and disease severity of study populations. Supplementary methods and variable definitions are provided in Supplementary Note 3 and Supplementary Table 4. Associations between study outcomes and potential effect modifiers were modeled on the entire corpus through univariate logistic regression assuming random-effects per study. Models were subsequently adjusted for medical condition, research question, and DMO domain and significance tests were adjusted through a Benjamini–Hochberg procedure^142^. In a sensitivity analysis, unreported study characteristics were treated as missing and multiple imputed using the method of chained equations and assuming the missing-at-random hypothesis^97,145^. Data analysis was conducted in R (version 3.6.1)^146^.

### Qualitative appraisal of the evidence

We defined a systematic qualitative appraisal protocol to synthesize and interpret our maps. For each medical condition, we compiled and appraised evidence related to DMOs’ known-groups validity, convergent validity, predictive validity, responsiveness, and ecological validity. Each DMO’s overall rating in each condition describes its potential for further validation as a clinical endpoint according to current evidence. The appraisal protocol is provided in Supplementary Tables 5 and 6.

### Consulting with relevant stakeholders

Levac et al. recommend that research teams involve stakeholders throughout the review process, as stakeholders can provide nuanced insights beyond those reported in the literature^107^. We regularly discussed review design and data interpretation with clinical, technical, epidemiological, regulatory, academic, and industry subject matter experts. Patients were not directly involved in this review. However, we plan to use these results in future priority-setting exercises with patient representatives.

### Supplementary information

Supplementary Materials

Supplementary Data 1

## Data Availability

The datasets generated during and/or analyzed during the current study are available in the OSF project repository (https://osf.io/k7395).

## Code availability

The code used to analyze included study data is available in the OSF project repository (https://osf.io/k7395).
