# Supplementary material for: Walking on common ground: a cross-disciplinary scoping review on the clinical utility of digital mobility outcomes
Source: NPJ Digit Med. 2021 Oct 14;4:149. doi: 10.1038/s41746-021-00513-5 (PMC8516969; doi:10.1038/s41746-021-00513-5)
Supplement: Supplementary file 1 — Supplementary Materials [file 41746_2021_513_MOESM1_ESM.pdf]

Supplementary Materials: Walking on common ground: A cross-disciplinary scoping review on the clinical utility of digital mobility outcomes

Table of Contents

Supplementary Review Methods **2**

Supplementary Table 1: Search strategy used in Medline 2

Supplementary Note 1: Definitions of Walking and Digital Mobility Outcomes 3

Supplementary Table 2: Operational definitions of key concepts adopted for this review 3

Supplementary Table 3: Narrative definitions of included digital mobility outcomes. 4

Supplementary Note 2: Review Materials and Reference Sheets 6

Supplementary Note 3: Assessing Risk of Bias 10

Supplementary Table 4: Definitions of variables used in meta-regression 10

Supplementary Note 4: Qualitative Appraisal - Methods 12

Supplementary Table 5: Definitions for qualitative appraisal 12

Supplementary Table 6: Overall ratings for qualitative appraisal 12

Supplementary Results **13**

Supplementary Table 7: Reasons for excluding records from this review 13

Supplementary Table 8: Characteristics of included records and study populations 14

Supplementary Figure 1: Number of eligible studies evaluating each DMO in the four included conditions. 14

Supplementary Note 5: Assessment of Bias - Results 15

Supplementary Figure 2: Associations between age and condition severity in the four included conditions 15

Supplementary Figure 3: Percentage of studies reporting significant outcomes, stratified into 20 quantiles. 16

Supplementary Table 9: Effects of study characteristics on the likelihood of significant study outcomes. 17

Qualitative Appraisal **18**

Supplementary Table 10: Qualitative Appraisal: Parkinson's Disease 18

Supplementary Table 11: Qualitative Appraisal: Multiple Sclerosis 19

Supplementary Table 12: Qualitative Appraisal: Chronic Obstructive Pulmonary Disease 20

Supplementary Table 13: Qualitative Appraisal: Proximal femoral fracture. 21

Systematic Maps **22**

Supplementary Note 6: Systematic Maps 22

Supplementary Figure 4: Known-groups validity – Differences between disease severity strata. 23

Supplementary Figure 5: Convergent validity - Associations between DMOs and measures of lower extremity function. 24

Supplementary Figure 6: Convergent validity - Associations between DMOs and balance measures. 25

Supplementary Figure 7: Convergent validity - Associations between DMOs and falls or fear of falling. .. 26

Supplementary Figure 8: Convergent validity - Associations between DMOs and measures of health-related quality of life. 27

Supplementary Figure 9: Convergent validity - Associations between DMOs and measures of health-related quality of life. 28

Supplementary Figure 10: Predictive validity of DMOs in Parkinson's disease. 29

Supplementary Figure 11: Responsiveness of DMOs used as primary or secondary endpoints in all eligible interventional studies. 30

Supplementary Figure 12: Ecological validity of DMOs in Parkinson's disease: Relationships between disease severity and DMOs collected in clinical vs real-world environments. 31

Supplementary References **32**

Mobilise-D Consortium Members **33**

Supplementary Review Methods

Supplementary Table 1: Search strategy used in Medline

#	Searches	Results
1	exp Pulmonary Disease, Chronic Obstructive/ or exp Parkinson Disease/ or exp Parkinsonian Disorders/ or exp Multiple Sclerosis/ or exp Demyelinating Diseases/ or exp Hip Fractures/ or (((chronic or lung or pulmonary or respirat* or airway* or airflow*) adj3 obstruct*) or copd).ti,ab. or (parkinson* or "paralysis agitans").ti,ab. or (((multipl* or disseminated or insular) adj3 scleros*) or "chariot disease" or demyelinat*).ti,ab. or ((hip* or femur* or femoral or trochant* or pertrochant* or intertrochant* or subtrochant* or intracapsular* or extracapsular*) adj5 fracture*).ti,ab.	403,875
2	(((step* or stride*) adj2 (speed or velocit* or time* or length* or width* or frequenc* or rate* or rhythm* or variabilit* or symmetr* or asymmetr* or count* or number* or distance* or cadence*)) or ((swing* or stance* or "single support" or "double support") adj2 (time* or duration* or variabilit* or symmetr* or asymmetr*)) or ((spatiotemporal or "spatio-temporal") adj2 (parameter* or feature* or characteristic*)) or ((gait or walk* or ambulat*) adj2 (speed or velocit* or time* or cadence* or pace* or rhythm* or volume* or bout* or duration* or distance* or intensit* or variabilit* or asymmetr* or symmetr* or parameter* or feature* or characteristic* or assess* or examin* or analys* or batter* or measure* or test*))).ti,ab.	80887
3	1 and 2	6890
4	limit 3 to yr="1999 - Current"	6358

The search strategy was used in Medline and similar searches were used in other databases. All search strategies used in each of the 11 queried databases are provided on our OSF project repository (https://osf.io/k7395)

Supplementary Note 1: Definitions of Walking and Digital Mobility Outcomes

Because understanding of seemingly common terms differs across disciplines, defining the concepts addressed by this review was not trivial. Therefore, our operational definitions of key concepts such as 'mobility', 'walking', 'real-world' and 'digital mobility outcomes' (DMOs) were defined via an adapted Delphi process[1] (Table S2). We pre-specified a list of included DMOs (Table S3), to include in this review. Pre-defined lists were defined by internal panels of clinical, technical, and research experts. Theoretically, DMOs could include any digital measures encompassed by the ICF definition of 'mobility.'[2] However, our scope was limited to a set of DMOs associated with walking, since walking is the primary focus of the Mobilise-D project. This list was compiled in consultation with mobility experts, technologists and clinicians in the four conditions. It includes spatiotemporal parameters and measures of daily volume of walking.

This list excludes non-linear gait and dynamic stability measures, such as Lyapunov exponents[3,4] and detrended fluctuation analyses,[5] as well as those based on the power spectral density analysis,[6] due to the emergent nature of their evidence base. Though we also consider digital measures of physical activity to be DMOs, physical activity measures indirectly related to walking, such as daily energy expenditure or activity intensity, are also out of scope. This is because physical activity represents a related, yet broader construct than walking.[7–9]

For ease of interpretation, DMOs were organized into the previously established categories Pace, Rhythm, Phase, Base of Support, Variability, and Symmetry.[10–13] DMOs in these categories are known to exhibit similar characteristics and are highly inter-correlated. Step count, walking time, walking bout length or duration were categorized as "Volume of Walking."

Supplementary Table 2: Operational definitions of key concepts adopted for this review

Concept	Maps generated in this review
Mobility	According to the International Classification of Functioning, Disability and Health (ICF), 'mobility' is defined as moving by (a) changing body position or location or by transferring from one place to another, (b) by carrying, moving or manipulating objects, (c) by walking, running or climbing and (d) by using various forms of transportation.[2]
Walking	Human walking is a method of locomotion and is defined as initiating and maintaining a forward displacement of the centre of mass in an intended direction involving the use of the two legs which provide both support and propulsion. The feet are repetitively and reciprocally lifted and set down whereby at least one foot is in contact with the ground at all times.[14,15] Walking with walking aids is included in this definition. *Walking* is made up of walking bouts and is equivalent to taking steps/stepping forward (thus stepping in place does not constitute walking) and is defined as starting from initial contact for the initial step until ending with full floor contact of the foot making the last step.[16]
Real-world Walking	'Real world' relates to the context in which walking takes place—that is free-living, unsupervised, uncontrolled and non-standardised. As such, it is unscripted as there are no instructions to the subject. Real-world actions occur in non-simulated everyday situations in unconstrained environments with minimal consciousness of being tested. It is equivalent to actions at home or in the community over continuous periods of time.[17] … Real-world walking is distinct from laboratory-based,[18] supervised (fully controlled and observed) and semi-controlled (walking 'freely' but with supervision) tests. It also is different from scripted or instructed walking, which can take place in the home or lab.
Digital mobility outcomes	Digital mobility outcomes are digitally-measured mobility parameters used to assess an individual's health status, such as spatiotemporal gait parameters, walking bout characteristics and physical activity. In this case, 'digital' measures refer to those objectively derived from electronic systems, as opposed to qualitative, paper-based or self-reported measures.

Supplementary Table 3: Narrative definitions of included digital mobility outcomes

DMO	Definition
**Pace**	
Walking speed	The distance covered by the whole body within a certain time interval or per unit time of walking.[15]
Step/Stride Length	Steps are typically defined as the anterior-posterior distance from the heel of one footprint to the heel of the opposite footprint.[13] For the purposes of this review, step length may also be measured between the toes or other identifiable markers on opposite footfalls. Stride length is equal to the length of two consecutive steps. These measures are known to be highly correlated and were combined for the purposes of this review.
**Rhythm**	
Cadence	The number of steps per minute, sometimes also referred to as step rate or frequency.[13]
Step/Stride Time	Step Time is the time elapsed from initial contact of one foot to initial contact of the opposite foot, while stride time is time elapsed between the initial contacts of two consecutive footfalls of the same foot.[13] These measures are known to be highly correlated and were combined for the purposes of this review.
**Phase**	
Stance time	The stance phase is the weight bearing portion of each gait cycle initiated at heel contact and ending at toe off of the same foot; stance time is the time elapsed between the initial contact and the last contact of a single footfall.[13]
Swing time	The swing phase is initiated with toe off and ends with initial contact of the same foot; swing time is the time elapsed between the last contact of the current footfall to the initial contact of the next footfall of the same foot.[13]
Single support time	Single support occurs when only one foot is in contact with the ground. Single support time is the time elapsed between the last contact of the opposite footfall to the initial contact of the next footfall of the same foot.[13]
Double support time	Double support occurs when both feet are in contact with the ground simultaneously; double support time is the sum of the time elapsed during two periods of double support in the gait cycle.[13]
**Base of Support**	
Step width	The lateral distance from heel center of one footprint to the line of progression formed by two consecutive footprints of the opposite foot.[13] For the purposes of this review, step width may also be measured between the toes or other identifiable markers on opposite footfalls.
Step width variability	Variability of step width, usually measured by standard deviation or coefficient of variation.
**Variability**	
Step/stride speed variability	Variability of step or stride speed during a walking bout, usually measured by standard deviation or coefficient of variation. Stride speed is the distance covered by the whole body within a single stride per unit time of walking.
Step/stride time variability	Variability of step or stride time during a walking bout, usually measured by standard deviation or coefficient of variation.

Step/stride length variability	Variability of step or stride length during a walking bout, usually measured by standard deviation or coefficient of variation.
Stance Time Variability	Variability of stance time during a walking bout, usually measured by standard deviation or coefficient of variation.
Swing time variability	Variability of stance time during a walking bout, usually measured by standard deviation or coefficient of variation.
Single support time variability	Variability of single support time during a walking bout, usually measured by standard deviation or coefficient of variation.
Double support time variability	Variability of double support time during a walking bout, usually measured by standard deviation or coefficient of variation.
**Asymmetry***	
Step speed asymmetry	Asymmetry of step speed of the left and right legs during a walking bout.
Step time asymmetry	Asymmetry of the step time of the left and right legs during a walking bout.
Step length asymmetry	Asymmetry of the step length of the left and right legs during a walking bout.
Swing time asymmetry	Asymmetry of the swing time of the left and right legs during a walking bout.
Single support time asymmetry	Asymmetry of the single support time of the left and right legs during a walking bout.
**Volume**	
Daily step count	The number of steps made during a set period of time, such as a day or walking bout. A *step* is the interval between the initial contacts of the ipsi- and contralateral foot.[14]
Daily walking time	The amount of time spent walking during a set period of time. Walking is made up of walking bouts and is equivalent to taking steps/stepping forward and is defined as starting from initial contact for the initial step until ending with full floor contact of the foot making the last step.[16]
Number, duration, or distance of walking bouts	A ***walking bout*** (WB) is a walking sequence containing at least two consecutive strides of both feet (e.g. *R-L-R-L-R-L* or *L-R-L-R-L-R*).<br>Start and end of a walking bout are determined by a resting period or any other activity (non-walking period). The initial step of a WB follows a non-walking period and the final step precedes the next non-walking period.<br>Number of walking bouts refers to the number of observed walking bouts in a defined period of time, typically one day. Duration refers to the time elapsed during a walking bout. Distance refers to the distance covered during a walking bout.

*DMOs related to walking asymmetry were not often studied, and were grouped during analysis for brevity.
DMO: Digital mobility outcome

Supplementary Note 2: Review Materials and Reference Sheets

Representative checklists and reference sheets are provided here. The full set of reference sheets for each condition is provided in the project's OSF project repository (https://osf.io/k7395).

1. Abstract screening checklist

Mobilise-D

Mobilise-D Scoping Review: Abstract Screening Worksheet

Overview:

- This review will explore the potential of DMOs as clinical trial endpoint measures by identifying, existing evidence on their construct validity, prognostic value, and responsiveness to intervention
- Our four research questions aim to explore the following:
    - RQ1: The differences in GaWPs between target populations and healthy controls
    - RQ2: The relationship between GaWPs and traditional clinical measurements
    - RQ3: The prognostic value of GaWPs
    - RQ4: The use of GaWPs as endpoints in interventional studies

Question 1: Should this paper be included in full-text review? (YES or NO)

	**Questions to ask yourself:**	**YES or Unsure**	**NO**
A	Is the study on an **included population**?<br>*(human studies on Parkinson's, Multiple Sclerosis, COPD, hip fracture)*	Proceed	Discard
B	Does the study assess **gait speed, gait analysis or an included GaWP**?<br>- See reference sheet for list of included GaWPs<br>- Note that some clinical walking tests are included as measures of gait speed (4 meter walk, 10 meter walk, timed 25 foot walk, etc.) and others are not. See reference sheet for details	Proceed	Discard
C	Is the study an **included design**?<br>○ Included Designs:<br>▪ Observational<br>▪ Case-control (comparing diseased group vs. healthy group)<br>▪ Cohort<br>▪ Cross-sectional<br>▪ Longitudinal<br>▪ Interventional<br>○ Excluded Designs:<br>▪ Case study<br>▪ Case series (Series of case studies published together)<br>▪ Review paper	Proceed	Discard
D	Could the study **address one of our research questions?**<br>*(answer YES if any of the following apply)*<br>- **RQ1:** Could the study explore the *differences in DMOs/GaWPs between healthy controls and a target population*?<br>- **RQ2/RQ3:** Could the study *explore a relationship between DMOs/GaWPs and included measurements (RQ2) or outcomes (RQ3) in a target population?*<br>○ Relationships could be in the form of a correlation, empirical relationship, odds ratio, risk ratio, hazard ratio, prediction model, multivariate analysis, or other association measure<br>- **RQ4:** Does the study appear to be an *interventional study in a target population with a DMO/GaWP as an endpoint*?	Proceed	Discard
E	Are at least **5 individuals (or 20 events** for RQ3) included in the final analysis?	Proceed	Discard

		**YES**	**NO**
F	Are there any other **inclusion criteria** that the study **clearly does not meet**?	**Discard**	**Keep**

***If you are unsure, please be conservative and include the study in full-text review.*

2. Abstract Screening Reference Sheet

Mobilise-D Scoping Review Abstract Screen: Reference Sheet

Eligibility Criteria

Criterion	Keep	Discard
Population	PD, MS, Hip Fracture, COPD, heart failure<br>Mixed populations IF a sub-analysis was conducted	Animal Studies<br>All other human disease areas<br>Mixed populations with no sub-analysis
Study Aim	Studies an included Gait and Walking Parameter according to one of our research questions	Studies with no GaWP and/or which do not address a RQ
Study Design	Case-control, cross sectional, longitudinal, cohort, controlled trials, protocols (RQ4 only)	Case study, case series<br>Systematic review (or any review)
Technology	Basically any (sensors, stopwatch, speed gaits, instrumented walkways, video, optometric systems, etc.)<br>Specific clinical tests regardless of technology use (see below)	Pedometers
Setting	Any (home, clinical, lab-based)	NA

Minimum Dataset			
RQ1	5 patients per study arm included in the final analysis	RQ3	Age, sex, and disease severity are included as covariates<br>At least 20 recorded events of an outcome of interest
RQ2	5 patients included in the final analysis	RQ4	5 patients per study arm included in final analysis

Gait and Walking Parameters

Spatial Parameters
Step length (mean, variability, asym.)
Stride length (mean, variability)
Step width (mean, variability)

Temporal Parameters
Cadence (mean, variability)
Step time (mean, variability, asymmetry)
Stride time (mean, variability)
Stance time (mean, variability, asymmetry)
Swing time (mean, variability, asymmetry)
Single support time (mean, variability, asym.)
Double support time (mean, variability)

Spatiotemporal Parameters
Gait speed (mean, variability)
Stride speed (mean, variability)

Volume of Walking
Walking time
Step count (excluding pedometer)
Number, length, duration of walking bouts

Included Walking Conditions

Keep:

- Gait analysis or measurement of any included gait parameters
- Dual-task walking, if testing scenario is included
- Some clinical tests, even no technology was used:
  - 4, 5, 10, 30, 50, etc. meter walk tests (or other short distance) – INCLUDE as gait speed
  - Timed 25 Foot Walk (T25FW) - INCLUDE as gait speed
  - 2 Minute Walk Test – INCLUDE as gait speed

Conditionally Keep:

- Timed Up & Go: ONLY INCLUDE instrumented TUG w/ GaWPs measured during walk
- Treadmill Walking:
  - Fixed-Speed Treadmill: INCLUDE any GaWP EXCEPT gait speed
  - Self-Adjusting Speed Treadmill: INCLUDE any GaWP
- 6Minute WT, 12Minute WT, 400m WT, (or other long walking tests):
  - Non-Instrumented Test: EXCLUDE
  - Instrumented test: INCLUDE any GaWP EXCEPT gait speed

Keep if normal gait may have been be analyzed at baseline or if walking condition was used as intervention (generally keep to be conservative):

- Tandem walking or other abnormal walking patterns
- Walking in time to cues (e.g., beats, music, beeping, etc.)
- Purposefully altering gait (e.g., instructions to concentrate on lifting toes)

Research Questions

RQ1: Comparison of GAWPs between a Mobilise-D population and healthy controls
RQ2: Association between a GaWP and a clinical measurement at a single timepoint
RQ3: Prognostic value: Longitudinal association between a GaWP and a clinical measurement or outcome over time
RQ4: Use of GaWPs as endpoints in controlled interventional studies

Population Terms

	Keep	Discard
PD	Parkinson('s) disease, Parkinsonism, idiopathic Parkinson's disease	Atypical parkinsonian syndromes, drug-induced parkinsonism, vascular parkinsonism, progressive supranuclear palsy, multiple system atrophy, corticobasal syndrome, dementia with lewy bodies
MS	Multiple Sclerosis, relapsing-remitting, primary progressive, secondary progressive MS	
PFF	Hip, femoral, intracapsular (subcapital and transcervical), extracapsular (trochanteric, intertrochanteric, pertrochanteric and subtrochanteric) fractures	Non-fracture-related hip arthroplasty or hip replacement
COPD	Chronic obstructive pulmonary disease<br>Chronic obstructive lung disease<br>Chronic respiratory disease (>age of 65)	Pulmonary hypertension,<br>Studies only including asthma patients

- *If the population is mixed, the study must conduct a sub-analysis of one of our populations to be included. If this is unclear from the abstract, be conservative and include.*
- *If you are unsure about the population and the paper meets all other inclusion criteria, include the paper and the disease-specific team will make the determination.*

FAQs

What do I do when…

I am not sure whether a measurement or outcome is included in RQ2/3 criteria

- Some determinations may require disease-specific knowledge. Be conservative and keep the paper.

A testing scenario or type of study is not covered by our eligibility criteria

- If something is not covered by eligibility criteria, raise a question to Ashley and others in the group. We may need to clarify an unforeseen situation.

The abstract does not indicate the technology or method used to measure a GaWP/DMO?

- Include the paper IF it mentions measuring an included gait parameter, gait analysis, or included clinical test of gait speed AND IF it meets all of our other inclusion criteria

Something is completely unclear, and I can't tell whether to include?

- Think about the item that is unclear with regard to the other inclusion criteria. How realistic is it that the criterion is met, given the information that you have?
- Be pragmatic, but inclusive. If all else fails, be conservative and keep the paper.

3. Example Full-text screening reference sheet: Parkinson's group

Mobilise-D Scoping Review – Full Text – Parkinson's Disease – General Eligibility Criteria

1. Research Questions

RQ1: Comparison of GAWPs between a Mobilise-D population and healthy controls
RQ2: Association between a GaWP and a clinical measurement at a single timepoint
RQ3: Prognostic value: Longitudinal association between a GaWP and a clinical measurement or outcome over time
RQ4: Use of GaWPs as endpoints in controlled interventional studies

2. General Eligibility Criteria

Criterion		Include	Exclude
2.1 Pub. Type		Peer-reviewed literature, Grey literature, Conference papers, PhD theses	Master's theses<br>Protocols (will be indexed for RQ4)<br>Clinical trial registration (indexed for RQ4)<br>Papers with no original results<br>Reviews & Meta-analyses
2.2 Study Design	RQ1	Case-control, cross sectional study, cross-sectional analysis of a longitudinal study,	Animal studies<br>Case studies<br>Case series<br>Systematic or non-systematic reviews<br>Protocols (will be indexed for RQ4)
	RQ2	Cross sectional study, cross-sectional analysis of a longitudinal study (inc. cohort studies, baseline analyses of controlled or uncontrolled interventional studies, etc.)	
	RQ3	Longitudinal (cohort) study or longitudinal analysis of the control arm of an interventional study	
	RQ4	Randomized or non-randomized controlled trials, including comparator trials and crossover design trials	RQ4: Uncontrolled interventional trials
2.3 Technology		Basically any (sensors, stopwatch, speed gaits, instrumented walkways, video, optometric systems, pedometers, etc.)<br>Specific clinical tests regardless of technology use (see below)	Self-reported daily activity/steps
2.4 Setting		Any (home, clinical, lab-based, inpatient, outpatient, indoor, outdoor)	NA
2.5 Minimum Dataset		>= 10 patients per study arm included in the final analysis	<10 patients per arm in any relevant analysis

3. Population Eligibility Criteria

Include	Exclude
-Confirmed diagnosis of Parkinson's disease by a professional physician based on the relevant diagnostic criteria at the time of the study's publication<br>-Any age range and disease severity level will be included	Animal studies<br>Atypical parkinsonian syndromes, drug-induced parkinsonism, vascular parkinsonism, progressive supranuclear palsy, multiple system atrophy, corticobasal syndrome, dementia with lewy bodies

- *If the population is mixed, the study must conduct a sub-analysis of one of our populations to be included.*

4. Gait and Walking Parameters

Temporal Parameters
Cadence (mean, variability)
Step time (mean, variability, asymmetry)
Stride time (mean, variability)
Stance time (mean, variability, asymmetry)
Swing time (mean, variability, asymmetry)
Single support time (mean, variability, asym.)
Double support time (mean, variability)

Spatial Parameters
Step length (mean, variability, asym.)
Stride length (mean, variability)
Step width (mean, variability)

Spatiotemporal Parameters
Gait speed (mean, variability)
Stride speed (mean, variability)

Volume of Walking
Daily Walking time
Daily Step count
Number, length, duration of walking bouts

Other
Gait variability (i.e., through autocorrelation or other methods))

5. Eligible Walking Conditions

5.1 - Included walking conditions:

- Gait analysis or measurement of any included gait parameter(real world, lab, or clinic)
- Single-task, double-task, straight, curvilinear, with or without turns, with or without obstacle avoidance
- Some clinical tests, even if no technology other than a stopwatch was used:
  - 4, 5, 10, 30, 50, etc. meter walk (or other short distance) – INCLUDE as gait speed
  - Timed 25 Foot Walk (T25FW) - INCLUDE as gait speed
  - 2 Minute Walk Test – INCLUDE as gait speed

5.2 - Conditionally Included:

- Timed Up & Go: ONLY INCLUDE instrumented TUG w/ GaWPs measured during walk
- Treadmill Walking:
  - Fixed-Speed Treadmill: INCLUDE any GaWP EXCEPT gait speed
  - Self-Adjusting Speed Treadmill: INCLUDE any GaWP
- 3/4/6/12Minute WT, 400m WT, (or other long walking tests):
  - Non-Instrumented Test: EXCLUDE
  - Instrumented test: INCLUDE any GaWP EXCEPT test time/average gait speed

5.3 - Excluded Walking Conditions (If GaWPs are measured ONLY in these conditions)

- Walking in time to cues (e.g., beats, music, beeping, mental singing, etc.)
- Walking during gait training (e.g., during a VR or auditory stimulation intervention)
- Walking during a condition/test designed to induce FoG
- *If a condition you encounter is unclear, raise a question to the group*

5.4 - Excluded Walking Motions (If GaWPs are measured ONLY during these motions)

- Turning, gait initiation, gait termination, stair climbing, during FoG, response to sudden stimulus or push, or other specific subsets of walking tasks
- Tandem walking, wide step walking, or other *intentional* non-natural walking Purposefully altering gait (e.g., instructions to concentrate on lifting toes)
- *If a condition you encounter is unclear, raise a question to the group*

Note: Papers studying excluded conditions and motions could still be included if normal gait was analyzed at baseline (RQ1/2) or as an outcome after intervention (RQ4)

6. RQ 1 Eligibility Criteria

Included Methods

- GaWPs meeting all other eligibility criteria are compared between a target population and a healthy population

7. RQ 2 Eligibility Criteria

Included Methods

- Establish the relationship between an included measure and a GaWP at a single timepoint
- Correlations, trends over stratified analyses, odds ratios, and other association measures are included

Included Measures

	Category		A - All disease areas	B – PD-specific Instruments	
1	Disease Severity & Symptoms		CGI (clinician global impression), PGI (patient global impression)	(MDS)-UPDRS – total, I, III, IV H&Y, RDRS, UDysRS, FoGQ, nFoGQ	1
2	Functional Status/ADL		Barthel Index, Nottingham EADL IADL, LLFDI, FIM	(MDS)-UPDRS – II, Schwab & England, SPDDS, SPES, PROMIS, Neuro-QoL, GNDS, Neuro rating scale from Scripps	2
3	HRQoL		EQ-5D (5L or 3L), EQ-VAS, SF-36 (RAND) SF-12, SF-MCS, SF-PCS, HUI3, LSQ	PDQ (8, 10, or 39)	3
4	Mental Health (Depression, Anxiety, Apathy)		HADS, Beck, CES-D, GDS, SDS/Zung, PHQ (2, 8, or 9), MHI, GHQ	LARS	4
5	Cognition		MMSE, MoCA, SDMT PASAT, CANTAB, CAMCOG-R	RBANS, ACE-R, PD-CRS, Trail Making Test, Digit Span, Stroop Color and Word Test	5
6	Falls		FES-I, Incidence of falls		6
7	Physical Function	Walking or Functional Assessments	6MWT, TUG, STS, SPPB (Total score)		7
8		Motor Function & Balance	ABC, Berg Balance, FAB, BESTest, mini-BESTest, Ambulation Index (AI)	360 degree (fast) turn test	8
9		Physical Activity	IPAQ, PASE, GLTEQ		9
10		Strength	Knee flexion (hamstring), Knee extension (Quadriceps), Leg press, Grip		10
11		Fatigue	FIS, mFIS, FSS, FACIT, U-FIS	PFS-16	11

Further information on included measures are included in the protocol supplemental materials

9. RQ 4 Eligibility Criteria

Included Methods

- Controlled studies, including comparator and crossover studies
- A GaWP is used as a primary, secondary, or exploratory endpoint

Excluded Methods

- Uncontrolled Studies
- Studies where an included GaWP is not measured as an outcome
- No new data available (protocols and registrations will be indexed but not analyzed)

8. RQ 3 Eligibility Criteria

8.1 - Included Methods

- Univariate analyses, prediction models, multivariate analyses, machine learning models, etc. that assess the relationship between a GaWP at baseline and an outcome at follow-up.
- Include retrospective or prospective analyses (if GAWP is assessed prior to outcome)

8.2 - Excluded Methods

- Models based on cross-sectional data (these belong to RQ2)
- Prediction models where gait speed/walking volume is the outcome

8.3 - General Outcomes: All disease areas

- Disease/Disability Status or Progression
- Health-Related Quality of Life
- Mortality
- Healthcare Utilization (e.g., hospitalizations, readmissions, home care, costs, invasive procedures, etc.)
- Physical Function (e.g., exercise capacity, motor function, balance, strength)
- Functional Status (e.g., activities of daily living)
- Fatigue
- Cognition
- Mental Health (e.g., depression, anxiety, apathy)
- Falls
- Life Space
- Residential Status
- Use of Mobility Aids

8.4 - PD-Specific Outcomes

- Development of Dyskinesia
- Development of Freezing of Gait
- Dopaminergic medication use
- Development of postural instability
- Dementia

Further information on included outcomes are included in the protocol supplemental materials

Supplementary Note 3: Assessing Risk of Bias

We assessed clinically-plausible sources of bias and effect modification in our corpus through manual inspection and random-effects meta-regression.[19–21] Potential effect modifiers included the speed and length of walking bouts, statistical analysis methods, and the size, median age and disease severity of study populations. Variable definitions are provided in Supplementary Table 4. Associations between study outcomes and potential effect modifiers were modeled on the entire corpus through univariate logistic regression assuming random effects per study. Models were subsequently adjusted for medical condition, research question, and DMO domain and significance tests were adjusted through a Benjamini-Hochberg procedure.[22] In a sensitivity analysis, unreported study characteristics were treated as missing and multiple imputed using the method of chained equations and assuming the missing-at-random hypothesis.[20,23] Data analysis was conducted in R (version 3.6.1).[24]

Supplementary Table 4: Definitions of variables used in meta-regression

Variable	Definition
Study Size	Number of participants included in the study<br>Continuous variable (scaled during analysis)
Age	Median age of the study population<br>Continuous variable (scaled during analysis)
Literature Type	Type of literature, variable collapsed into three levels: (1) Peer-reviewed literature, (2) Conference literature, or (3) all other gray literature
Speed	Describes the prescribed speed of a walking task<br>Categorical variable with 2 levels:<br>(1) Habitual: Participants were instructed to walk at a self-selected, habitual speed<br>(2) Fast: Participants were instructed to walk at a fast or top speed
Length	Describes the length of a walking task used in a study<br>Categorical variable with 2 levels:<br>(1) Short: Less or equal to than 20m in length or 1 minute in duration<br>(2) Long: More than 20m in length or 1 minute in duration
Cognitive Load	Describing the cognitive load during the walking task, either as Single vs Dual-Task Walking<br>Categorical variable with 2 levels:<br>(1) Single Task: Task was conducted under single-task conditions<br>(2) Dual Task: Task was conducted under any dual-task condition
Severity*	Median disease severity of the study population<br>Categorical variable with 3 levels: (1) mild, (2) moderate, (3) severe conditions according to cut points established in the literature:

	Mild	Moderate	Severe
Parkinson’s disease			
UPDRS-III	<=32	>32 to <59	59+
Hoehn & Yahr	<2	2 to <3.5	3.5+
Multiple Sclerosis			
EDSS	<3	3 to <5.5	5.5+
PDDS	<2	2 to <3.5	3.5+
Chronic obstructive pulmonary disease			
FEV_1 % predicted	80+	>30 to <80	<=30
GOLD Stages (1-4)	<2	2 to <3.5	3.5+

	Proximal Femoral Fracture			
	Gait Speed	>0.8 m/s	>0.4 to 0.8 m/s	<=0.4 m/s
Risk*	Notes whether the analysis was conducted on specific sub-population at risk for gait impairment, including fallers, people with freezing of gait, severe disease, etc.<br>Categorical variable with 2 levels:<br>(1) General population: The analysis was not conducted on a specific sub-population,<br>(2) At-Risk population: The analysis was conducted on specific sub-population at risk for gait impairment, including fallers, people with freezing of gait, etc.			
Matched	Notes whether healthy controls and patients were matched for age, sex, or height in the known-groups validity analysis.<br>Categorical variable with 2 levels: (1) Unmatched, (2) Matched			
Analysis Method	Statistical analysis method used to assess cross-sectional (RQ2) or longitudinal (RQ3) relationships between DMOs and clinically-relevant measures or outcomes.<br>Categorical variable with 3 levels:<br>(1) Bivariate: Crude associations between two measures, generally through a correlation coefficient<br>(2) Stratified: Differences identified between stratified groups, such as disease severity strata, fallers vs. non-fallers, etc., via parametric or nonparametric hypothesis tests<br>(3) Multivariate: Associations identified through multivariate or adjusted models			
Research Question	The research question (RQ) addressed by this analysis. RQ1: Differences between patients and healthy controls; RQ2: Cross-sectional associations between DMOs and clinically-relevant measures; RQ3: Predictive validity of DMOs - relationships between DMOs at baseline and future outcomes; RQ4: Responses of DMOs to intervention in controlled clinical trials			
Domain	Domains of DMOs studied in the review<br>Categorical variable with 7 levels: (1) Pace, (2) Rhythm, (3) Phases, (4) Base of support, (5) Variability, (6) Symmetry, (7) Volume			
Condition	Conditions studied in the review<br>Categorical variable with 4 levels: (1) Parkinson’s disease, (2) multiple sclerosis, (3) chronic obstructive pulmonary disease, or (4) proximal femoral fracture			

*During analysis, Severe condition severity and At-risk populations were combined due to a limited number of observations of study populations with severe conditions.

DMO: Digital Mobility Outcome, RQ: Research Question, UPDRS: Unified Parkinson's disease rating scale, EDSS: Expanded disability status scale, FEV_1%pred: Percentage of predicted forced expiratory volume in 1 second

Supplementary Note 4: Qualitative Appraisal - Methods

The following protocol was used to qualitatively appraise the evidence for each DMO:

1. Rate evidence as ++, +, -, or ? for each psychometric property and (if applicable) measurement category individually, according to the definitions in Supplementary Table 5.

Supplementary Table 5: Definitions for qualitative appraisal

**Known-groups validity, Convergent validity, Responsiveness**	
++	Bernoulli test is statistically significant, more than 10 records were identified, and the proportion of studies with significant results is >0.7
+	Bernoulli test is significant, and:<br>• proportion of studies with significant results is between 0.3-0.7 OR<br>• fewer than 10 records were identified
?	Unable to rate from available data. Bernoulli test is not significant and <5 records identified.
-	Five or more records were identified AND<br>• the Bernoulli test is not significant OR<br>• the proportion of studies with significant results is less than 0.3
**Prognostic value**	
++	Five or more records were identified and the majority of records show significant results
+	Two or more records identified and the majority of records show significant results
?	Unable to rate from available data
-	Two or more records were identified. The majority of records show negative results
**Ecological validity**	
++	Five or more records were identified. Trends are similar to in-clinic findings.
+	One or more records were identified. Trends are similar to in-clinic findings
?	Unable to rate from available data.
-	Three or more records were identified. Trends differ from in-clinic findings

2. Compile evidence in each of five categories (Ecological validity, known-groups validity, convergent validity, prognostic value, responsiveness) by adding the number of + in each category. If only negative evidence exists, the category is assigned -1. If no evidence or unclear evidence exists, the category is assigned "?". Responsiveness should only be evaluated in studies which exhibited efficacy via positive primary outcomes.

3. Rate the evidence for each DMO according to the definitions in Supplementary Table 6

Supplementary Table 6: Overall ratings for qualitative appraisal

**Overall Ratings**	
++	4-5 categories have positive evidence
+	2-3 categories have positive evidence
?	Unable to rate from available data. Evidence is conflicting or too little evidence was identified
-	Only negative evidence was identified

Supplementary Results

Supplementary Table 7: Reasons for excluding records from this review

Reasons for Exclusion	n (%)*
The study did not address one of our research questions	1435 (71.0)
Only excluded gait parameters were studied	608 (29.7)
DMOs were assessed, but only during gait initiation, turns, stair climbing, or other excluded walking motions/conditions	109 (5.3)
Fewer than 10 participants were included in any relevant analysis	268 (13.1)
The study population did not meet our inclusion criteria	138 (6.7)
Part of the study population met our criteria, but a sub-analysis on these participants was not conducted	126 (6.2)
No included measurements, instruments, or outcomes were studied	202 (9.9)
The record studied prognostic value, but looked at DMOs as outcomes rather than variables	31 (1.5)
The record reported an interventional study, but it was uncontrolled and no other relevant analysis was reported	242 (11.8)
The study design was a case study, case series, review, master's thesis, or other non-eligible study type	112 (5.5)
The record was an interventional protocol that used a DMO as an outcome that otherwise meets the criteria for RQ4	154 (7.5)
The record was a poster or conference abstract with insufficient information reported to know if a relevant analysis was conducted	834 (40.7)
The record reported identical results as another record, but to a different conference or journal	278 (13.6)
Full text was not available<br>• Not found following multi-national library search (n=1; Potentially a fake citation, as the journal was discontinued prior to the publication date)<br>• Thesis or other record type under an embargo period (n=7)	8 (0.4)
Full text was not available one the included languages (English, German, French, Spanish, Catalan, Portugese, Italian, Norwegian, Swedish, Danish, Russian). Excluded languages were Turkish(n=4), Arabic (n=2), Farsi (n=1), Japanese (n=7), and Mandarin (n=16).	30 (1.5)

*Records were often excluded for multiple reasons, and summed percentages exceed 100%

Supplementary Table 8: Characteristics of included records and study populations

	PD<br>n = 307	MS<br>n = 270	COPD<br>n = 225	PFF<br>n = 53
**Record Characteristics**				
Peer-Reviewed Article	256 (83.4%)	206 (76.3%)	155 (68.9%)	48 (90.6%)
Letter to the Editor	3 (1.0%)	1 (0.4%)	3 (1.3%)	0 (0.0%)
Conference Submission	36 (11.7%)	57 (21.1%)	62 (27.6%)	2 (3.8%)
Doctoral Thesis	8 (2.6%)	4 (1.5%)	3 (1.3%)	3 (5.7%)
Report	1 (0.3%)	1 (0.4%)	0 (0.0%)	0 (0.0%)
Other	3 (1.0%)	1 (0.4%)	2 (0.9%)	0 (0.0%)
**Population Characteristics**				
Number of Participants	42 [28 - 66]	54 [30 - 91]	69 [38 - 137]	65 [34 - 104]
Age (years)	66.7 [64.3 - 69.5]	47.6 [42.2 - 51.0]	67.0 [64.3 - 69.7]	80.0 [78.0 - 82.3]
Severity	UPDRS_III (n=261)<br>26.5 [20.0 - 31.7]	EDSS (n=243)<br>3.5 [2.5 - 4.5]	FEV_1 %pred (n=224)<br>50.0 [43.5 - 56.0]	Gait Speed (n=26)<br>0.6 [0.3 - 0.9]

Data are presented as n (%) of included records or median [interquartile range]
PD: Parkinson's disease, MS: Multiple sclerosis, COPD: Chronic obstructive pulmonary disease, PFF: proximal femoral fracture; UPDRS: Unified Parkinson's disease rating scale, EDSS: Expanded disability status scale, FEV_1%pred: Percentage of predicted forced expiratory volume in 1 second

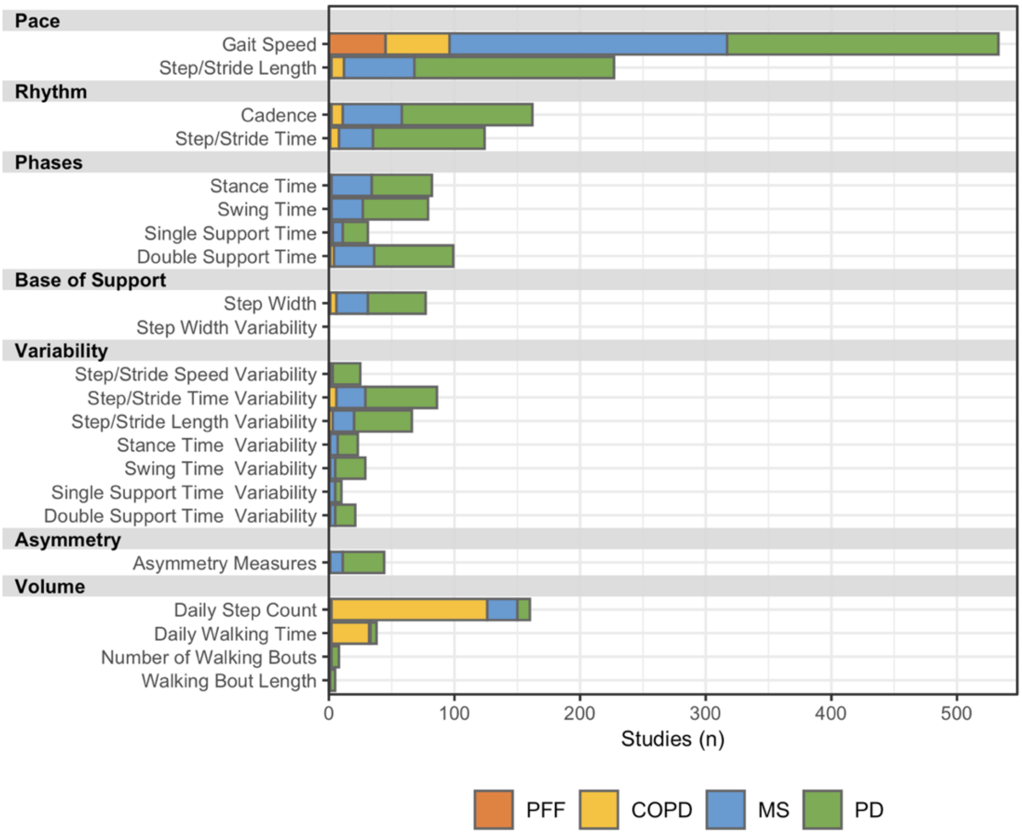

Supplementary Figure 1: Number of eligible studies evaluating each DMO in the four included conditions.
DMOs known to be highly inter-correlated were grouped (i.e., step length and stride length), and all DMOs were organized according to previously established domains of gait.
PD: Parkinson's disease, MS: Multiple sclerosis, COPD: Chronic obstructive pulmonary disease, PFF: proximal femoral fracture

Supplementary Note 5: Assessment of Bias - Results

Theoretically, disease severity should be positively associated with age. This relationship only appears to exist in studies on multiple sclerosis, which represented a younger population (Supplementary Figure 2). The body of literature on PD, COPD, and PFF appears to exhibit a survivorship bias (in this case, the tendency for healthier-than-average individuals with a given characteristic to be included in a study) with respect to age and condition severity. This is likely due to an association between age, condition severity, and co-morbidities or cognitive impairment, which were often exclusion criteria in included studies. Upon manual inspection, larger studies appear more likely to report significant findings than smaller studies (Supplementary Figure 3). This trend was supported in univariate associations between study size and study outcomes, but was no longer significant following adjustment for medical condition, research question, and DMO domain.

Meta-regression showed that conference abstracts, studies with fast walking assessments, and studies on at-risk subgroups such as fallers were more likely to report significant results than their counterparts. Conversely, studies on populations with mild disease severity, were less likely to report significant findings than those with moderate severity. In studies comparing pathological to healthy gait, those that matched patients and controls for gait speed were less likely to report significant findings for any DMO. In studies investigating the prognostic value of DMOs, adjusted models were more likely to yield significant findings than univariate analyses, suggesting potential publication bias. No other study characteristics were associated with study outcomes. Sensitivity analyses yielded similar estimates of all effects.

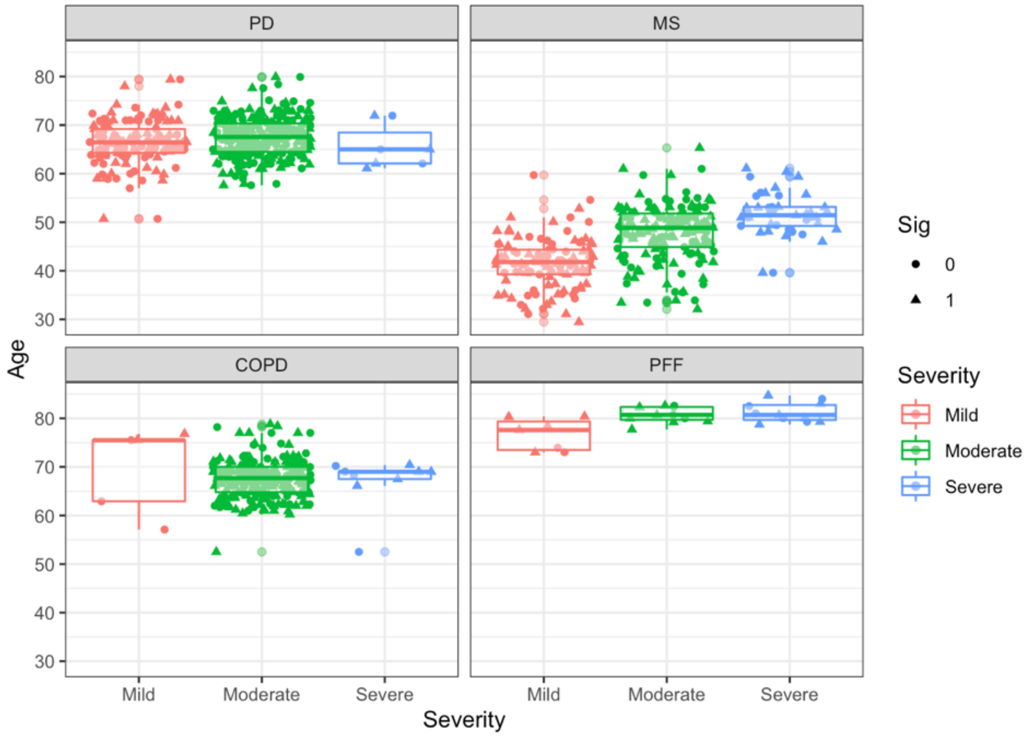

Supplementary Figure 2: Associations between age and condition severity in the four included conditions

PD: Parkinson's disease, MS: Multiple sclerosis, COPD: Chronic obstructive pulmonary disease, PFF: proximal femoral fracture, Sig: Analysis results were significant

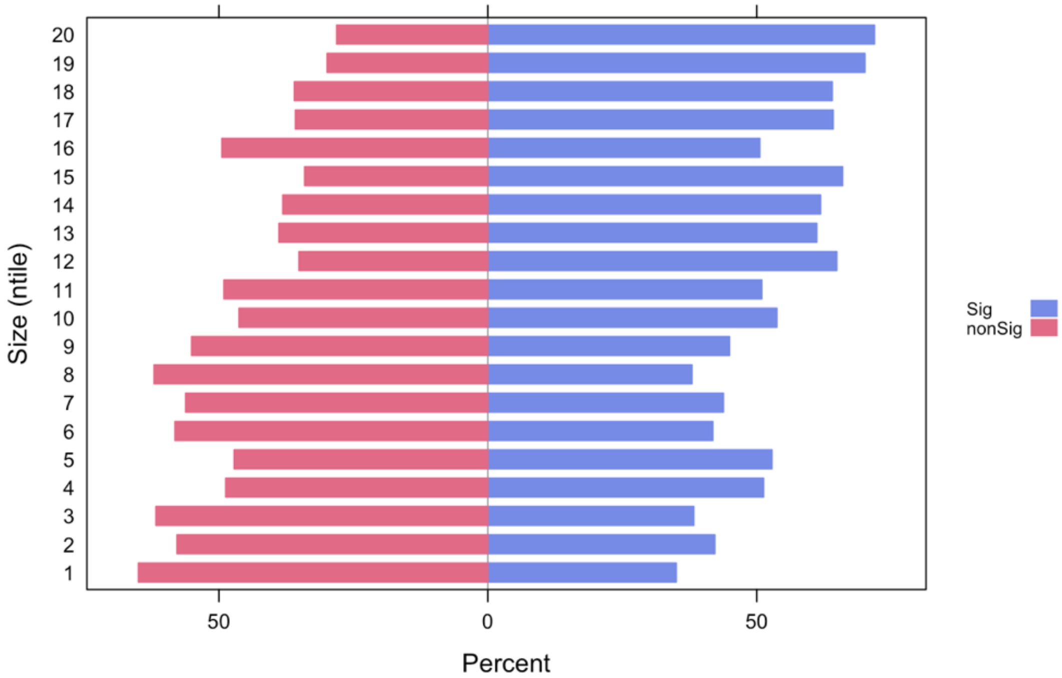

Supplementary Figure 3: Percentage of studies reporting significant outcomes, stratified into 20 quantiles.

Sig: Analysis results were significant, nonSig: Analysis results were not significant

Supplementary Table 9: Effects of study characteristics on the likelihood of significant study outcomes

Study Characteristic	Unadjusted models		Adjusted Models	
	Primary Analysis	Sensitivity Analysis	Primary Analysis	Sensitivity Analysis
**General variables**				
Size (Scaled)	1.28 [1.07 - 1.52]*	1.27 [1.07 - 1.52]*	1.11 [0.98 - 1.26]	1.11 [0.98 - 1.26]
Age (Scaled)	0.85 [0.72 - 1.01]	0.88 [0.71 - 1.1]	1.34 [0.94 - 1.91]	1.13 [0.79 - 1.63]
Conference (vs. peer-reviewed) records	3.54 [2.31 - 5.44]*	3.54 [2.31 - 5.44]*	2.44 [1.59 - 3.76]*	2.45 [1.59 - 3.76]*
Other unreviewed (vs. peer-reviewed) records	1.02 [0.59 - 1.77]	1.02 [0.59 - 1.77]	1.12 [0.63 - 2.02]	1.13 [0.63 - 2.02]
Fast (vs. self-selected) speed	2.25 [1.66 - 3.06]*	1.64 [1.25 - 2.17]*	1.54 [1.1 - 2.17]*	1.35 [1.01 - 1.79]
Long (vs. short) walking bout length	1.07 [0.81 - 1.43]	1.07 [0.82 - 1.4]	1.25 [0.91 - 1.7]	1.24 [0.9 - 1.7]
Dual-task (vs. single-task) walking	0.86 [0.64 - 1.16]	0.86 [0.64 - 1.16]	0.96 [0.7 - 1.33]	0.96 [0.7 - 1.33]
Mild (vs. moderate) condition severity	0.46 [0.35 - 0.6]*	0.51 [0.4 - 0.66]*	0.46 [0.34 - 0.61]*	0.49 [0.38 - 0.64]*
At-risk (vs. general) population	1.86 [1.37 - 2.53]*	1.86 [1.37 - 2.53]*	2.03 [1.47 - 2.8]*	2.03 [1.47 - 2.8]*
**Research question (RQ) specific variables**				
RQ1: Matched (vs. unmatched) for age, sex, or height	0.86 [0.55 - 1.34]	0.86 [0.55 - 1.34]	0.81 [0.49 - 1.35]	0.81 [0.49 - 1.35]
RQ1: Controlled (vs. uncontrolled) for gait speed	0.46 [0.23 - 0.93]*	0.31 [0.15 - 0.63]*	0.39 [0.18 - 0.83]*	0.37 [0.17 - 0.82]*
RQ2: Stratified (vs. univariate) analysis	0.82 [0.56 - 1.19]	0.84 [0.57 - 1.23]	1 [0.68 - 1.48]	1.03 [0.68 - 1.54]
RQ2: Multivariate (vs. univariate) analysis	0.88 [0.51 - 1.53]	0.94 [0.54 - 1.64]	0.95 [0.54 - 1.67]	1.01 [0.56 - 1.79]
RQ3: Adjusted (vs. univariate) analysis	3.18 [1.36 - 7.45]*	3.03 [1.28 - 7.15]*	2.71 [1.14 - 6.48]*	2.63 [1.1 - 6.31]*

Data are presented as Odds Ratio [95% Confidence Interval]. Adjusted models were adjusted for research question, medical condition, and digital mobility outcome domain.
*Significantly different from 1.0 following Benjamini-Hochberg correction for multiple testing

Qualitative Appraisal

Supplementary Table 10: Qualitative Appraisal: Parkinson's Disease

Psychometric Property	KGV	KGV	CV	CV	CV	CV	CV	CV	CV	CV	CV	PV	PV	PV	R	EV	
	vs. Healthy Controls	Severity Strata	Severity	Physical Function	HRQOL	ADL	Falls	Balance	Mental Health	Cognition	Freezing of Gait	Falls	Disease Progression	Physical Function	Responsiveness	Similar trends: clinic vs. real-world	Overall
Gait Speed	++	++	++	+	+	?	++	+	+	-	++	++	+	+	+	+	++
Step/Stride Length	++	+	++	+	?	?	++	++	?	-	+	?			+	+	++
Cadence	+	+	+	?	?		-	?		-	+		+	+	++	+	++
Step/Stride Time	+	-	+	?	?		+	?	?	?	+	-	-	-	-	+	+
Stance Time	+	+	+	?	+		?	+		?	?	?			+	+	++
Swing Time	+	+	+	?	?		?	?		?	?	-			?	+	+
Single Support Time	+	?	?							?	+						+
Double Support Time	+	?	+	?	?			+	?	?	+				-		+
Step Width	-	?	?	?		?	?	?		?	?	-			?		-
Step Width Variability	-	?	?				?	?		?	?				?		-
Step/Stride Speed Variability	+		?				?	?		?	+	-			?	+	+
Step/Stride Time Variability	+	?	+	?	+		+	+		?	?	?	+	+	-	+	++
Step/Stride Length Variability	+	+	+	?		?	+	?	?	-	+	-			-	+	+
Stance Time Variability	+	?	?				?			?	?	+			?	+	+
Swing Time Variability	+	?	?				?	?		?	?	?			?	+	+
Single Support Time Variability	+										?						?
Double Support Time Variability	+		?	?				?		?	?				?		?
Asymmetry Measures	+		+		?		?	?		?	+	?	-	+	-	+	++
Daily Step Count	?		?		+					?					+		+
Daily Walking Time	+	?	+	?	?		?	?	?						?		+
Number of Walking Bouts	?	?	?	?	?				?		?	-			?		-
Walking Bout Length	+	?	?								?	?					?
Walking Bout Length Variability	?	?	?														?

KGV – Known-groups validity, CV – Convergent Validity, PV – Predictive Validity, EV – Ecological Validity

Supplementary Table 11: Qualitative Appraisal: Multiple Sclerosis

Psychometric Property	KGV	KGV	CV	CV	CV	CV	CV	CV	CV	CV	CV	CV	CV	PV	PV	PV	R	EV	
	vs. Healthy Controls	Severity Strata	Severity	Physical Function	HRQOL	ADL	Falls	Balance	Mental Health	Strength	Cognition	Physiological Measures	Fatigue	Falls	Disease Progression	Activities of Daily Living	Responsiveness	Similar trends: clinic vs. real-world	Overall
Gait Speed	++	++	++	++	+	+	+	++	+	+	++	+	+	+	+	+	+	+	++
Step/Stride Length	++	+	+	+			+		?			?	?				+		+
Cadence	+	+	++	+		?	?	?	?				?				+	+	++
Step/Stride Time	+	+	+	+			+		?		?	?	?				?	+	+
Stance Time	+	+	+	+			?		?		?	?	?				?	+	+
Swing Time	+	+	+	+			?				?	?					?	+	+
Single Support Time	+	+	+	?									?				?		+
Double Support Time	++	+	++	+			?		?			?	?				?		+
Step Width	+	+	+	+			?		?				?				?		+
Step Width Variability	-	?	?				?										?		-
Step/Stride Speed Variability														-					?
Step/Stride Time Variability	++	+	+	?			+		?		?	?		+				?	+
Step/Stride Length Variability	++	?	+	?			+		?		?	?							+
Stance Time Variability	?	?	?	?			?					?						+	?
Swing Time Variability	+	+	+								?	?							+
Single Support Time Variability	?	?	?	?			+		?										+
Double Support Time Variability	?						?					?					?		-
Asymmetry Measures	+	+	+	?			?						?				?		+
Daily Step Count	+	+	++	++	?			?				+	+		+		?		+
Daily Walking Time		?	?																?
Number of Walking Bouts	?	?	?	?															?
Walking Bout Length	?			?															+

KGV – Known-groups validity, CV – Convergent Validity, PV – Predictive Validity, EV – Ecological Validity

Supplementary Table 12: Qualitative Appraisal: Chronic Obstructive Pulmonary Disease

Psychometric Property	KGV	KGV	CV	CV	CV	CV	CV	CV	CV	CV	CV	CV	PV	PV	PV	PV	R	EV	
	vs. Healthy Controls	Severity Strata	Severity	Physical Function	HRQOL	ADL	Falls	Balance	Mental Health	Strength	Exacerbations	Physiological Measures	Mortality	Disease Progression	Healthcare Utilization	Activities of Daily Living	Responsiveness	Similar trends: clinic vs. real-world	Overall
Gait Speed	++	+	+	++	++				+	+	?	+	++		?		?	+	++
Step/Stride Length	+	?	+	+						?							?		+
Cadence	+	+	+	?						?							?	+	+
Step/Stride Time	+	?	?							?									?
Stance Time	?	?	?							?									?
Swing Time	+	?	?							?									?
Single Support Time	?																		?
Double Support Time	+	?	?							?									?
Step Width	?	?	?							?									?
Step Width Variability	?		?																?
Step/Stride Speed Variability	?																		?
Step/Stride Time Variability	+	?	?	?															?
Step/Stride Length Variability	+		?																?
Stance Time Variability	?																		?
Swing Time Variability	?																		?
Double Support Time Variability	?																		?
Daily Step Count	++	++	++	++	+				+	+	+	+	++	+	?	?	+		++
Daily Walking Time	++	+	+	++	+				?	++		-			?		+		+

KGV – Known-groups validity, CV – Convergent Validity, PV – Predictive Validity, EV – Ecological Validity

Supplementary Table 13: Qualitative Appraisal: Proximal femoral fracture

Psychometric Property	KGV	KGV	CV	CV	CV	CV	CV	CV	CV	CV	CV	PV	PV	PV	R	EV	
	vs. Healthy Controls	Severity Strata	Severity	Physical Function	HRQOL	ADL	Falls	Balance	Mental Health	Strength	Cognition	Mortality	Healthcare Utilization	Activities of Daily Living	Responsiveness	Similar trends: clinic vs. real-world	Overall
Gait Speed	+		?	+	+	+	+	+	?	+	?		+	?	+		++
Step/Stride Length															?		?
Cadence															?		?
Step/Stride Time	?																?
Single Support Time															?		?
Double Support Time										?	?				?		?
Step Width															?		?
Step/Stride Speed Variability										?	?				?		?
Asymmetry Measures										?	?				?		?
Daily Step Count				?			?	?		?					?		?

KGV – Known-groups validity, CV – Convergent Validity, PV – Predictive Validity, EV – Ecological Validity

Systematic Maps

Supplementary Note 6: Systematic Maps

Supplementary Figures 4-11 present additional systematic maps, including constructs and analyses not shown in the main paper, as well as evidence divided by study setting (clinic/lab vs. real-world). Some studies included both in-clinic and real-world measures, thus the number of studies in the clinic/lab maps plus the number of studies in the real-world maps may not equal the overall map.

Data are always presented as the Number of studies with statistically significant associations between DMOs and measures of lower extremity function / Total studies (%). DMOs known to be highly inter-correlated were grouped (i.e., step length and stride length), and all DMOs were organized according to previously established domains of gait.

Proportions noted with an asterisk (*) indicate that the proportion of studies with positive results exceeds the expected false positive rate as determined by Bernoulli hypothesis testing and Benjamini-Hochberg adjustment.

Acronyms:
PD: Parkinson's disease
MS: Multiple Sclerosis
COPD: chronic obstructive pulmonary disease
PFF: proximal femoral fracture
ADL: Activities of daily living

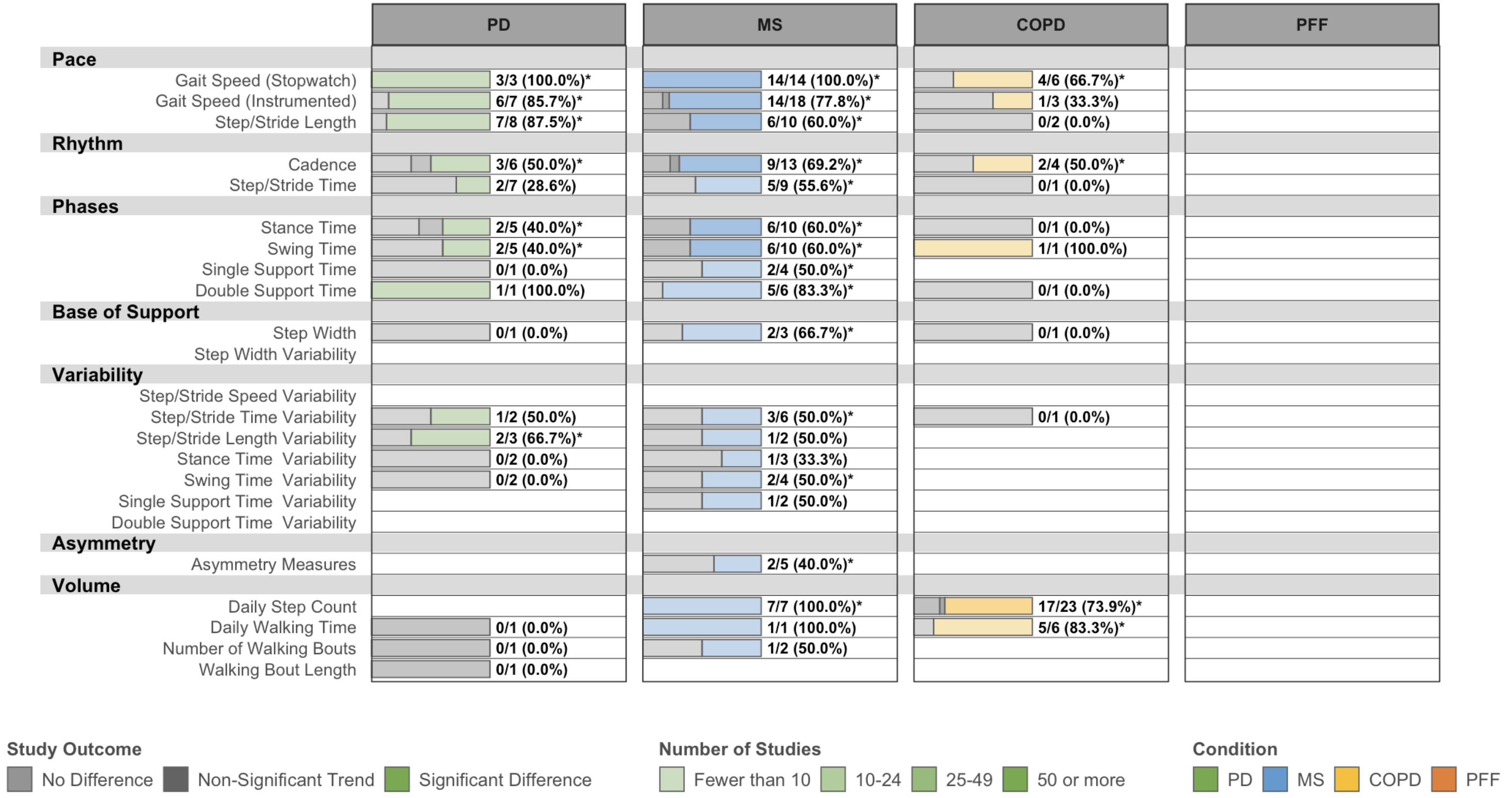

Supplementary Figure 4: Known-groups validity – Differences between disease severity strata. Data are presented as: Number of studies with statistically significant associations between DMOs and measures of disease severity / Total studies (%). Disease severity measures include the UPDRS, UPDRS-III, and Hoehn & Yahr scale in PD, EDSS and PDDS in MS, FEV_1% predicted and GOLD Stage in COPD, and patient or physician rated global measures of improvement in all four conditions. Most relevant measures in PFF fell under different categories, such as activities of daily living. DMOs known to be highly inter-correlated were grouped (i.e., step length and stride length), and all DMOs were organized according to previously established domains of gait.

*Proportion of studies exceeds the expected false positive rate as determined by Bernoulli hypothesis testing and Benjamini-Hochberg adjustment.

PD: Parkinson's disease, MS: Multiple Sclerosis, COPD: chronic obstructive pulmonary disease, PFF: proximal femoral fracture.

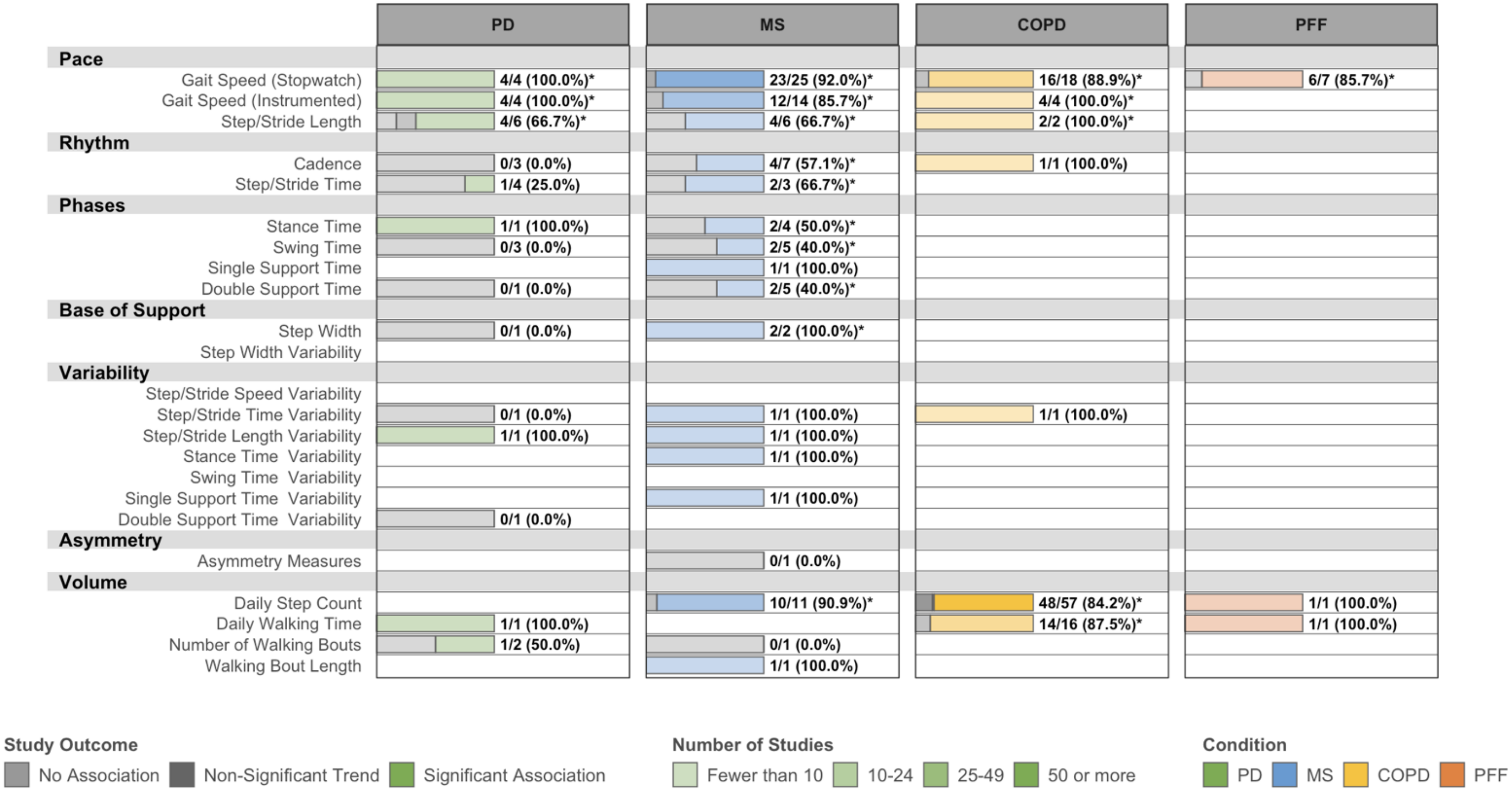

Supplementary Figure 5: Convergent validity - Associations between DMOs and measures of lower extremity function. Data are presented as: Number of studies with statistically significant associations between DMOs and measures of lower extremity function / Total studies (%). Measures included the Timed Up and Go, 6-minute walk test, incremental and endurance shuttle walk tests, and the short physical performance battery. DMOs known to be highly inter-correlated were grouped (i.e., step length and stride length), and all DMOs were organized according to previously established domains of gait.

*Proportion of studies exceeds the expected false positive rate as determined by Bernoulli hypothesis testing and Benjamini-Hochberg adjustment.

PD: Parkinson's disease, MS: Multiple Sclerosis, COPD: chronic obstructive pulmonary disease, PFF: proximal femoral fracture.

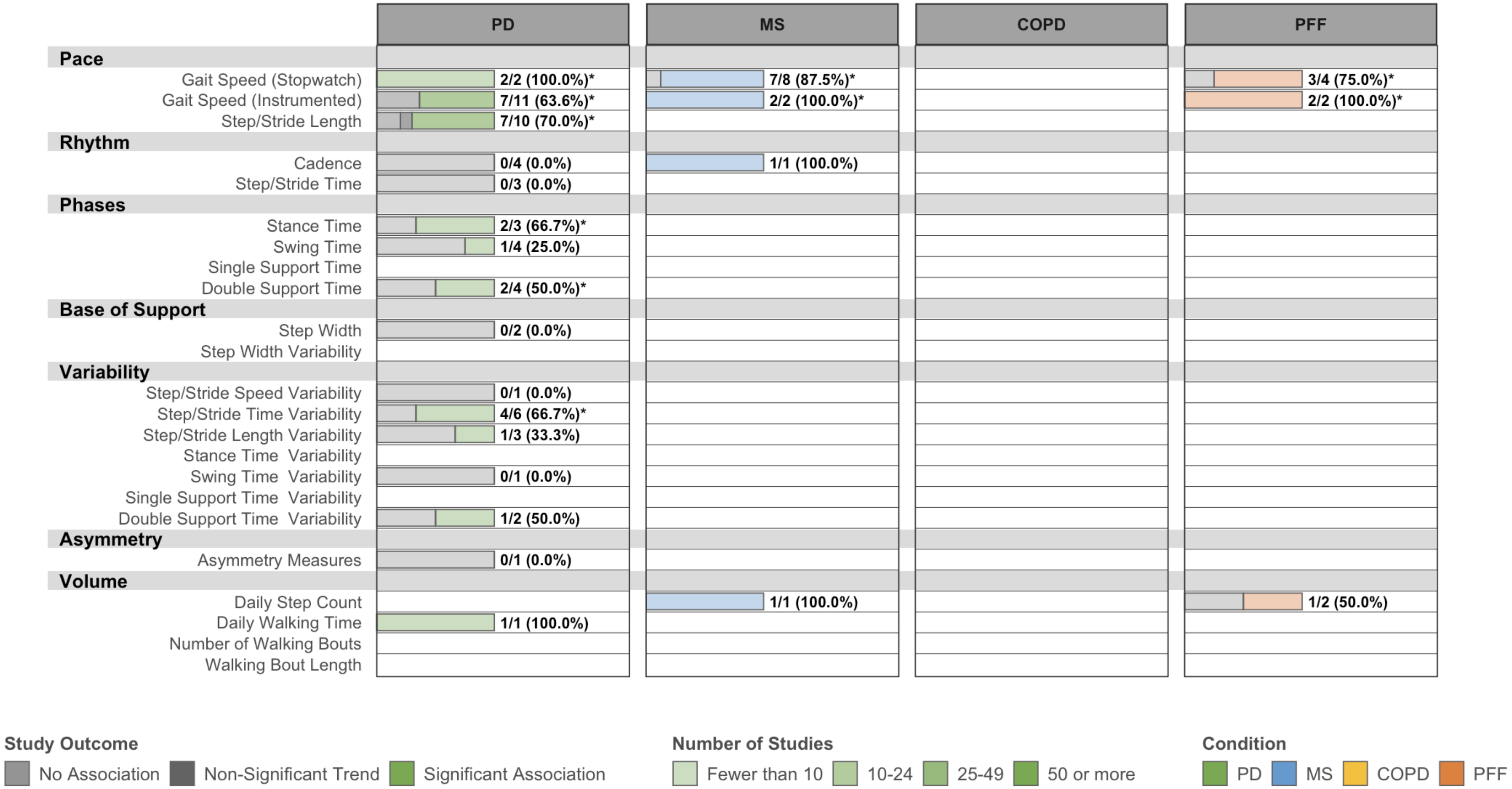

Supplementary Figure 6: Convergent validity - Associations between DMOs and balance measures. Data are presented as: Number of studies with statistically significant associations between DMOs and measures of lower extremity function / Total studies (%).DMOs known to be highly inter-correlated were grouped (i.e., step length and stride length), and all DMOs were organized according to previously established domains of gait.

*Proportion of studies exceeds the expected false positive rate as determined by Bernoulli hypothesis testing and Benjamini-Hochberg adjustment.

PD: Parkinson's disease, MS: Multiple Sclerosis, COPD: chronic obstructive pulmonary disease, PFF: proximal femoral fracture.

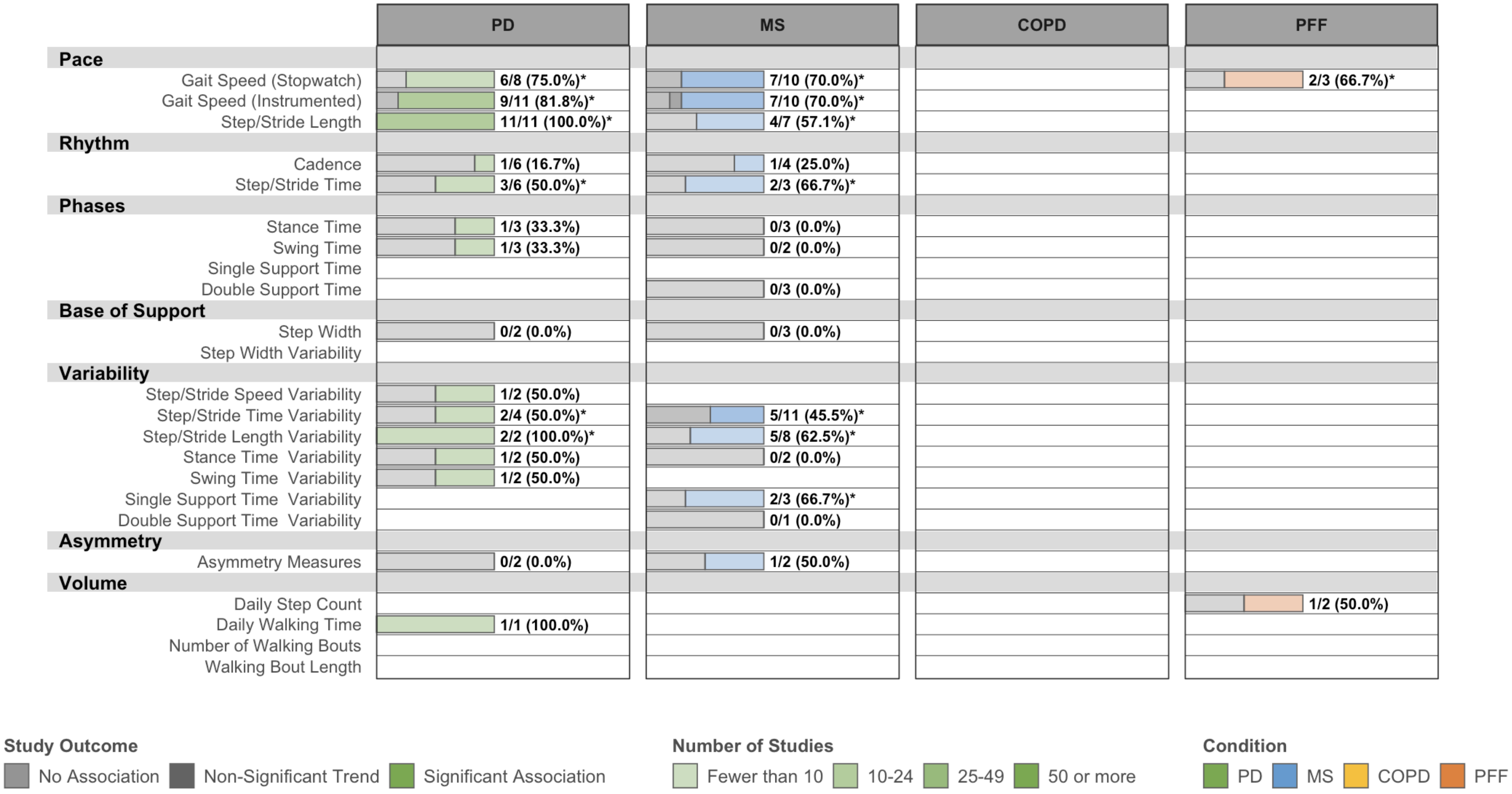

Supplementary Figure 7: Convergent validity - Associations between DMOs and falls or fear of falling. Data are presented as: Number of studies with statistically significant associations between DMOs and measures of lower extremity function / Total studies (%).DMOs known to be highly inter-correlated were grouped (i.e., step length and stride length), and all DMOs were organized according to previously established domains of gait.

*Proportion of studies exceeds the expected false positive rate as determined by Bernoulli hypothesis testing and Benjamini-Hochberg adjustment.

PD: Parkinson's disease, MS: Multiple Sclerosis, COPD: chronic obstructive pulmonary disease, PFF: proximal femoral fracture.

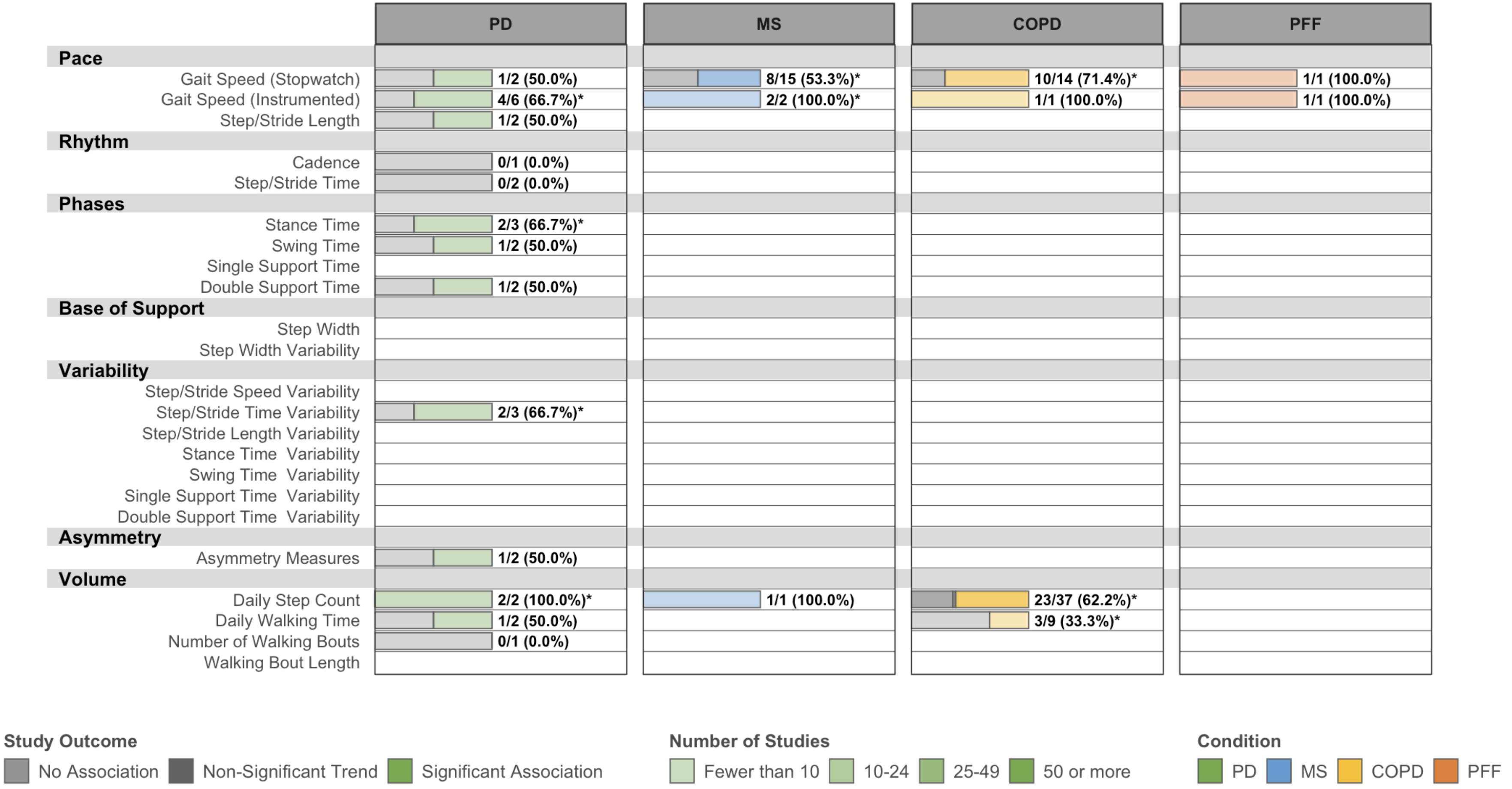

Supplementary Figure 8: Convergent validity - Associations between DMOs and measures of health-related quality of life. Data are presented as: Number of studies with statistically significant associations between DMOs and measures of lower extremity function / Total studies (%). DMOs known to be highly inter-correlated were grouped (i.e., step length and stride length), and all DMOs were organized according to previously established domains of gait.
*Proportion of studies exceeds the expected false positive rate as determined by Bernoulli hypothesis testing and Benjamini-Hochberg adjustment.
PD: Parkinson's disease, MS: Multiple Sclerosis, COPD: chronic obstructive pulmonary disease, PFF: proximal femoral fracture.

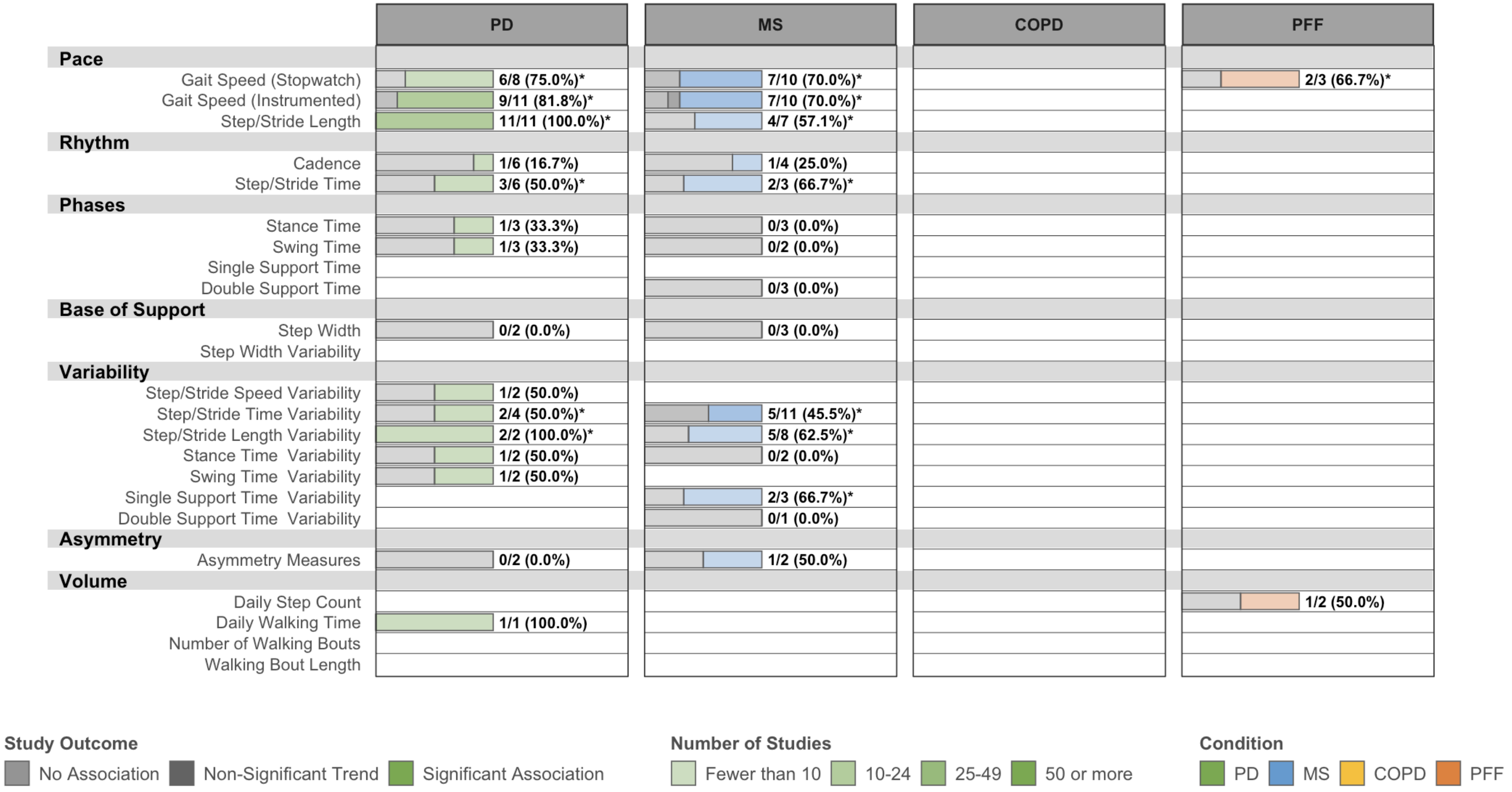

Supplementary Figure 9: Convergent validity - Associations between DMOs and measures of health-related quality of life. Data are presented as: Number of studies with statistically significant associations between DMOs and measures of lower extremity function / Total studies (%). DMOs known to be highly inter-correlated were grouped (i.e., step length and stride length), and all DMOs were organized according to previously established domains of gait.

*Proportion of studies exceeds the expected false positive rate as determined by Bernoulli hypothesis testing and Benjamini-Hochberg adjustment.

PD: Parkinson's disease, MS: Multiple Sclerosis, COPD: chronic obstructive pulmonary disease, PFF: proximal femoral fracture.

	Disease progression	Falls	Cognition	Physical function
**Pace**				
Gait Speed (Stopwatch)		1/1 (100.0%)		1/1 (100.0%)
Gait Speed (Instrumented)	2/2 (100.0%)	2/3 (66.7%)	2/3 (66.7%)	1/1 (100.0%)
Step/Stride Length		1/3 (33.3%)	2/3 (66.7%)	
**Rhythm**				
Cadence	1/1 (100.0%)			1/1 (100.0%)
Step/Stride Time	0/1 (0.0%)	0/1 (0.0%)		0/1 (0.0%)
**Phases**				
Stance Time		1/2 (50.0%)	1/2 (50.0%)	
Swing Time		0/1 (0.0%)	0/1 (0.0%)	
Single Support Time			1/2 (50.0%)	
Double Support Time			1/2 (50.0%)	
**Base of Support**				
Step Width		0/1 (0.0%)	1/1 (100.0%)	
Step Width Variability				
**Variability**				
Step/Stride Speed Variability		0/1 (0.0%)		
Step/Stride Time Variability	1/1 (100.0%)	1/3 (33.3%)	1/2 (50.0%)	1/1 (100.0%)
Step/Stride Length Variability		0/1 (0.0%)	1/2 (50.0%)	
Stance Time Variability		2/2 (100.0%)	1/1 (100.0%)	
Swing Time Variability		1/3 (33.3%)	1/1 (100.0%)	
Single Support Time Variability				
Double Support Time Variability				
**Asymmetry**				
Asymmetry Measures	0/1 (0.0%)	1/2 (50.0%)	1/1 (100.0%)	1/1 (100.0%)
**Volume**				
Daily Step Count				
Daily Walking Time				
Number of Walking Bouts		0/1 (0.0%)		
Walking Bout Length		1/2 (50.0%)		

Study Outcome: No Association; Non-Significant Trend; Significant Association

Number of Studies: Fewer than 10; 10-24; 25-49; 50 or more

Supplementary Figure 10: Predictive validity of DMOs in Parkinson's disease. Data are presented as: Number of studies with statistically significant associations between DMOs and measures of lower extremity function / Total studies (%). DMOs known to be highly inter-correlated were grouped (i.e., step length and stride length), and all DMOs were organized according to previously established domains of gait.

*Proportion of studies exceeds the expected false positive rate as determined by Bernoulli hypothesis testing and Benjamini-Hochberg adjustment.

PD: Parkinson's disease, MS: Multiple Sclerosis, COPD: chronic obstructive pulmonary disease, PFF: proximal femoral fracture.

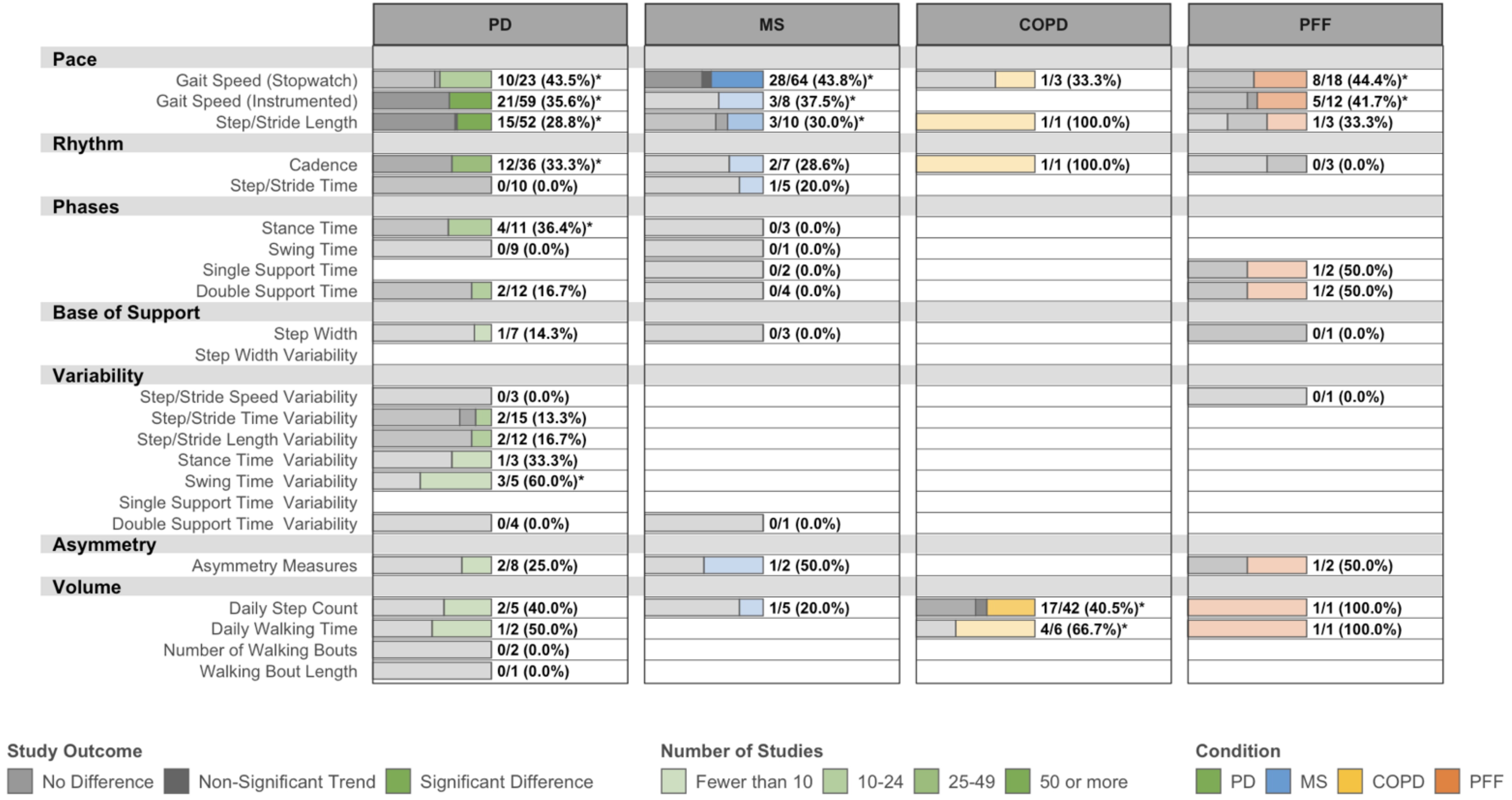

Supplementary Figure 11: Responsiveness of DMOs used as primary or secondary endpoints in all eligible interventional studies. Data are presented as: Number of studies with statistically significant differences between groups / Total studies (%). Interventions in eligible studies were not necessarily effective, and this map may underestimate the responsiveness of DMOs. DMOs known to be highly inter-correlated were grouped (i.e., step length and stride length), and all DMOs were organized according to previously established domains of gait.

*Proportion of studies exceeds the expected false positive rate as determined by Bernoulli hypothesis testing and Benjamini-Hochberg adjustment.

PD: Parkinson's disease, MS: Multiple Sclerosis, COPD: chronic obstructive pulmonary disease, PFF: proximal femoral fracture.

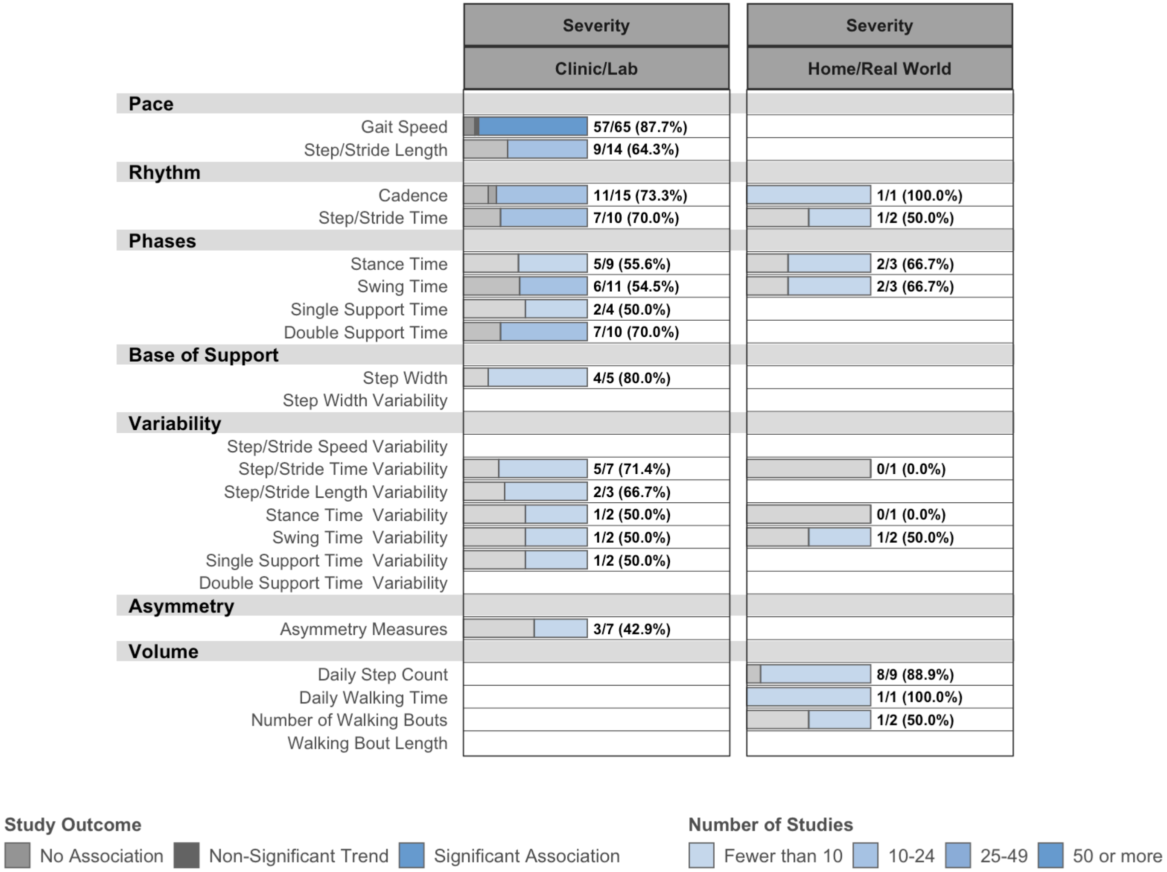

Supplementary Figure 12: Ecological validity of DMOs in Parkinson's disease: Relationships between disease severity and DMOs collected in clinical vs real-world environments. Data are presented as: Number of studies with statistically significant associations between DMOs and measures of lower extremity function / Total studies (%). DMOs known to be highly inter-correlated were grouped (i.e., step length and stride length), and all DMOs were organized according to previously established domains of gait.

*Proportion of studies exceeds the expected false positive rate as determined by Bernoulli hypothesis testing and Benjamini-Hochberg adjustment.

PD: Parkinson's disease, MS: Multiple Sclerosis, COPD: chronic obstructive pulmonary disease, PFF: proximal femoral fracture.

Supplementary References

1. Kluge, F. *et al.* Consensus based framework for digital mobility monitoring. Preprint available at https://www.medrxiv.org/content/10.1101/2020.12.18.20248404v2. (2020).
2. International Classification of Functioning, Disability and Health (ICF). Available at: https://www.who.int/standards/classifications/international-classification-of-functioning-disability-and-health. (Accessed: 6th May 2021)
3. England, S. A. & Granata, K. P. The influence of gait speed on local dynamic stability of walking. *Gait Posture* **25**, 172–178 (2007).
4. Buzzi, U. H., Stergiou, N., Kurz, M. J., Hageman, P. A. & Heidel, J. Nonlinear dynamics indicates aging affects variability during gait. *Clin. Biomech.* **18**, 435–443 (2003).
5. Dingwell, J. B. & Cusumano, J. P. Re-interpreting detrended fluctuation analyses of stride-to-stride variability in human walking. *Gait Posture* **32**, 348–353 (2010).
6. Weiss, A. *et al.* Toward automated, at-home assessment of mobility among patients with Parkinson disease, using a body-worn accelerometer. *Neurorehabil. Neural Repair* **25**, 810–818 (2011).
7. Dlugonski, D., Wójcicki, T. R., McAuley, E. & Motl, R. W. Social cognitive correlates of physical activity in inactive adults with multiple sclerosis. *Int. J. Rehabil. Res.* **34**, 115–120 (2011).
8. Fakolade, A. *et al.* Understanding leisure-time physical activity: Voices of people with MS who have moderate-to-severe disability and their family caregivers. *Heal. Expect.* **21**, 181–191 (2018).
9. Dobbels, F. *et al.* The PROactive innovative conceptual framework on physical activity. *Eur. Respir. J.* **44**, 1223–1233 (2014).
10. Thingstad, P. *et al.* Identification of gait domains and key gait variables following hip fracture. *BMC Geriatr.* **15**, 150 (2015).
11. Lahousse, L. *et al.* Gait patterns in COPD: the Rotterdam Study. *Eur. Respir. J.* **46**, 88–95 (2015).
12. Lord, S. *et al.* Independent Domains of Gait in Older Adults and Associated Motor and Nonmotor Attributes: Validation of a Factor Analysis Approach. *Journals Gerontol. Ser. A Biol. Sci. Med. Sci.* **68**, 820–827 (2013).
13. Hollman, J. H., McDade, E. M. & Petersen, R. C. Normative spatiotemporal gait parameters in older adults. *Gait Posture* **34**, 111–118 (2011).
14. Perry, J. *Ganganalyse: Norm und Pathologie des Gehens*. (Urban & Fischer, 2003).
15. Whittle, M. *Gait analysis: An introduction*. (Butterworth-Heinemann, 2005).
16. Dijkstra, B., Kamsma, Y. P. & Zijlstra, W. Detection of gait and postures using a miniaturized triaxial accelerometer-based system: Accuracy in patients with mild to moderate Parkinson's disease. *Arch. Phys. Med. Rehabil.* **91**, 1272–1277 (2010).
17. Del Din, S., Godfrey, A., Galna, B., Lord, S. & Rochester, L. Free-living gait characteristics in ageing and Parkinson's disease: Impact of environment and ambulatory bout length. *J. Neuroeng. Rehabil.* **13**, (2016).
18. Iluz, T. *et al.* Automated detection of missteps during community ambulation in patients with Parkinson's disease: A new approach for quantifying fall risk in the community setting. *J. Neuroeng. Rehabil.* **11**, 48 (2014).
19. Boutron, I. *et al.* Chapter 7: Considering bias and conflicts of interest among the included studies. in *Cochrane Handbook for Systematic Reviews of Interventions version 6.2* (eds. Higgins, J. et al.) (2021).
20. Deeks JJ, Higgins JPT, A. D. Chapter 10: Analysing data and undertaking meta-analyses. in *Cochrane Handbook for Systematic Reviews of Interventions version 6.2* (eds. Higgins, J. et al.) (2021).
21. Thompson, S. G. & Sharp, S. J. Explaining heterogeneity in meta-analysis: a comparison of methods. *Stat. Med.* **18**, 2693–2708 (1999).
22. Benjamini, Y. & Hochberg, Y. Controlling the False Discovery Rate: A Practical and Powerful Approach to Multiple Testing. *J. R. Stat. Soc. Ser. B* **57**, 289–300 (1995).
23. Donders, A. R. T., van der Heijden, G. J. M. G., Stijnen, T. & Moons, K. G. M. Review: A gentle introduction to imputation of missing values. *J. Clin. Epidemiol.* **59**, 1087–1091 (2006).
24. R Core Team. R: A language and environment for statistical computing. (2020).

Mobilise-D Consortium Members

Graham Armitage[1], Jaume Bacardit[1], Silvia Del-Din[1], Brook Galna[1], Ann Gibson[1], Harry Bailey[1], Hugo HIden[1], Sara Johansson Fernstad[1], Alma Cantu[1], Cameron Kirk[1], Encarna Micó Amigo[1], Lou Sutcliffe[1], Isabel Neatrour[1], Annette Pantall[1], Phil Brown[1], Lisa Alcock[1], Jian Qing Shi[1], Lynn Rochester[1], Lynda Speed[1], Paul Watson[1], Alison Yarnall[1], Tecla Bonci[2], Gavin Brittain[2], Daisy Priest[2], Sarah Birchall[2], Sarah Moll[2], Liam Haslam[2], Dibya Pattanaik[2], Alex Radford[2], Ahmed Mubarak-Mohamed[2], David Paling[2], Shivani Patel[2], Siva Nair[2], Sooji Han[2], Fabio Ciravegna[2], Neil Ireson[2], Vita Lanfranchi[2], Claudia Mazzà[2], Basil Sharrack[2], Kirsty Scott[2], Azza Ishmail[2], Ahmed Mubarak-Mohamed[2], Ellen Buckley[2], Linda Van Gelder[2], Lubos Vaci[2], Jeff Hausdorff[3], David Buzaglo[3], Marina Brozgol[3], Nir Giladi[3], Pablo Cornejo Thumm[3], Inbar Hillel[3], Eran Gazit[3], Ayala Saban[3], Anat Mirelman[3], Daniela Berg[4], Marie Bornhorst[4], Kirsten Emmert[4], Kristin Hansen[4], Clint Hansen[4], Hanna Hildesheim[4], Lisa-Marie Chidex[4], Katharina Dirksen[4], Vera Helmker[4], Lisa Marie Behncke[4], Elke Warmerdam[4], Janet Vanuem[4], Minh Hoang Pham[4], Corina Maetzler[4], Pia Reisdorf[4], Christian Schlenstedt[4], Walter Maetzler[4], Lorenzo Chiari[5], Giorgio Davico[5], Francesca Bottin[5], Roberta De Michele[5], Saverio Ranciati[5], Ilaria D'Ascanio[5], Giuliano Galimberti[5], Luca Reggi[5], Cristina Curreli[5], Luca Palmerini[5], Marco Viceconti[5], Heleen Demeyer[6], Nitesh Ghosh[6], Marieke Wuyts[6], Valerie Haerens[6], Femke Hulzinga[6], Lies Glorie[6], Pieter Ginis[6], Wim Janssenns[6], Thierry Troosters[6], Myriam Witvrouw[6], Sofie Breuls[6], Alice Nieuwboer[6], Kamiar Aminian[7], Sabine Schoendorf[7], Gilbert Buesching[7], Judith Peterhans[7], Thomas Riegler[7], Anisoara Ionescu[7], Abolfazl Soltani[7], Bjoern Eskofier[8], Felix Kluge[8], Arne Küderle[8], Sabine Stallforth[8], Sylvia Kraus[8], Martin Ullrich[8], Brian Caulfield[9], Tahar Kechadi[9], Alison Keogh[9], David Singleton[9], Cathy Goulding[9], Abhishek Bhattacharya[9], Georgiana Ifrim[9], Antonio Bevilaqua[9], Brian Mac Namee[9], Mara Diaconu[10], Anna Marcuzzi[10], Erika Skaslien[10], Mari Odden[10], Ingalill Midtsand[10], Monika Engdal[10], Kristin Bjornstadjordet[10], Mara Manailescu[10], Martin Aursand Berge[10], Karoline Blix Grønvik[10], Jorunn Helbostad[10], Lars Gunnar Johnsen[10], Hanne Stene Snerting[10], Ronny Bergquist[10], Ingvild Saltvedt[10], Kristin Taraldsen[10], Beatrix Vereijken[10], Clemens Becker[11], Simon Jaeger[11], Carl-Philipp Jansen[11], Martha Gierka[11], Christoph Endress[11], Carmen Lamparter[11], Niki Brenner[11], Clarissa Huber[11], Svenja Klaus-Karwisch[11], Bernd Kinner[11], Jochen Klenk[11], Stefanie Mikolaizak[11], Kilian Rapp[11], Matthias Schwab[11], Martin Wohlrab[11], Lars Schwickert[11], Stefano Bertuletti[12], Francesca Salis[12], Marco Caruso[12], Andrea Cereatti[12], Marta Benet Mora[13], Alicia Josa[13], Mariona Font Garcia[13], Gabriela Cardenas[13], Victoria Alcaraz Serrano[13], Ines Cobo[13], Magda Bosch de Basea[13], Anne-Elie Carsin[13], Anna Delgado Llobet[13], Laura Delgado Ortiz[13], Judith Garcia Aymerich[13], Elena Gimeno-Santos[13], Sarah Koch[13], Nikolaos Chynkiamis[14], Dimitrios Megaritis[14], Emily Hume[14], Ioannis Vogiatzis[14], Mike Polkey[15], Alexis Perkins[15], Parris Williams[15], Keir Philip[15], Sara Buttery[15], Nicholas Hopkinson[15], Comi Giancarlo[16], Lucia Roselli[16], Maria Libera La Porta[16], Matteo Martinis[16], Prete Federica[16], Battisti Raffaella[16], Gloria Dallacosta[16], Letizia Leocani[16], Anja Frei[17], Milo Puhan[17], Gilbert Buesching[17], Judith Peterhans[17], Sabine Schoendorf[17], Thomas Riegler[17], Melanie Keller[17], Marc Spielmanns[17], Thomas Sigrist[17], Katherine Hackl[17], Valerie Zumbrunnen[17], Ashley Polhemus[17], David Wenn[18], Michael Jackson[18], Jordi Evers[19], Neall Mouthaan[19], Siete Frouws[19], Martijn Niessen[19], Luca Cornelisse[19], Laura Siepman[19], Lucas Pluimgraaff[19], Rob van Lummel[19], Heiko Gassner[20], Andrea Weitzenfelder[20], Sarah Seifferth[20], Teresa Greinwalder[20], Franz Marxreiter[20], Jelena Jukic[20], Martin Regensburger[20], Juergen Winkler[20], Stafanie Dettmer[20], Sabine Stallforth[20], Jochen Klucken[20], Anne-Marie Kirsten[21], Ines Zimmermann[21], Kirsten Paash[21], Martina Russ[21], Henrik Watz[21], Hubert Blain[22], Sylvie Broussous[22], Francois Canovas[22], Florent Cerret[22], Louis Dagneaux[22], Valerie Driss[22], Florence Galtier[22], Stephanie Miot[22], Charlote Kaan[22], Anne-Sophie Vérissimo[22], Hajar Ahachad[22], Eva Murauer[22], Mats Ericson[23], Gilles Gnacadja[23], Lening Shen[23], Jules Desmond[23], Anja Kassner[23], Beat Knusel[23], Mary Elliot-Davey[23], Hoi-Shen Radcliffe[23], Magnus Jornten-karlsson[24], Jesper Havsol[24], Pierre Mugnier[24], Diana Jarretta[24], Solange Corriol Rohou[24], Gabriela Saraiva[24], Henrik Sillén[24], Michael Boettger[25], Igor Knezevic[25], Frank Kramer[25], Paolo Piraino[25], Stephen Raymond[26], Ara Sekaram[26], Patrick Folaron[26], Natalia Pletneva[26], Karin Beckstrom[26], Stefanie Krieger[26], Narcis Nica[26], Fay Horak[26], Kristen Sowalsky[26], Antonia Gizdic[26], Skender Imeri[26], Valdo Arnera[26], Ashar Ahmad[26], Marcel Froehlich[27], Paul Nolan[28], Barbara Skerrit[28], Michal Skackov[28], Gerard Quinn[28], Marie Mc Carthy[28], Aida Aydemir[29], Yann Hyvert[29], Wim Dartee[30],

Sabina Hernandez Penna[30], Tilo Hache[30], Oliver Grenet[30], Ram Miller[30], Arne Mueller[30], Jens Praestgaard[30], Sandra Schluechter[30], Gul Erdemli[30], Leen van Steenbergen[30], Ronenn Roubenoff[30], Shyamal Patel[31], Matt Czech[31], Isik Karahanoglu[31], Xuemei Cai[31], Charmaine Demanuele[31], Hao Zhang[31], Dimitrios Psaltos[31], Joe Mather[31], Anil Tarachandani[31], Nadir Ammour[32], Matthieu Jouannin[32], Manon Cariou[32], Mike Chambers[33], Ariel Dowling[33], Antonella Ciucchiuini[33], Sara Shnider[34], Mark Gordon[34], Lucy Fry[34], Michal Melamed[34], Pippa Loupe[34], Michael Reich[34]

1. University of Newcastle upon Tyne, Newcastle, United Kingdom
2. University of Sheffield, Sheffield, United Kingdom
3. The Foundation For Medical Research Infrastructural Development And Health Services, Tel Aviv, Israel
4. University of Kiel (Christian-Albrechts-Universität), Kiel, Germany
5. Alma Mater Studiorum - Università di Bologna, Bologna, Italy
6. Katholieke Universiteit Leuven, Leuven, Belgium
7. Ecole Polytechnique Federale de Lausanne, Lausanne, Switzerland
8. Friedrich-Alexander-Universitaet Erlangen-Nuernberg, Erlangen, Germany
9. University College Dublin, Dublin, Ireland
10. Norwegian University of Science and Technology, Trondheim, Norway
11. Robert-Bosch-Hospital, Stuttgart, Germany
12. University of Sassari, Sassari, Italy
13. IS Global, Barcelona, Spain
14. University of Northumbria at Newcastle, Newcastle, Italy
15. Imperial College of Science Technology and Medicine, London, United Kingdom
16. San Raffaele University, Milan, Italy
17. University of Zurich, Zurich, Switzerland
18. Ixscient Ltd, Middlesex, United Kingdom
19. McRoberts B.V., The Hague, Netherlands
20. University Hospital Erlangen, Erlangen, Germany
21. Pneumologisches Forschungsinstitut an der LungenClinic Grosshansdorf GmbH, Grosshansdorf, Germany
22. Centre Hospitalier Universitaire de Montpellier, Montpellier, France
23. Amgen Limited, Cambridge, United Kingdom
24. AstraZeneca AB, Gothenberg, Sweden
25. Bayer AG, Leverkusen, Germany
26. eResearch Technology Inc., Philadelphia, USA
27. Gruenenthal GMBH, Aachen, Germany
28. ICON Clinical Research Limited, Dublin, Ireland
29. Merck KGaA, Darmstadt, Germany
30. Novartis Pharma AG, Basel, Switzerland
31. Pfizer Limited, Sandwich, United Kingdom
32. Sanofi Aventis Research & Development, Chilly-Mazarin, France
33. Takeda, Tokyo, Japan
34. Teva Pharmaceutical Industries Ltd, Petah Tikva, Israel
